# Combinatorial metabolomic and transcriptomic analysis of muscle growth in hybrid striped bass (female white bass *Morone chrysops* x male striped bass *M. saxatilis*)

**DOI:** 10.1186/s12864-024-10325-y

**Published:** 2024-06-10

**Authors:** Sarah A. S. Rajab, Linnea K. Andersen, Linas W. Kenter, David L. Berlinsky, Russell J. Borski, Andrew S. McGinty, Christopher M. Ashwell, Peter R. Ferket, Harry V. Daniels, Benjamin J. Reading

**Affiliations:** 1 Department of Applied Ecology, North Carolina State University, 100 Eugene Brooks Avenue, Box 7617, Raleigh, NC 27695 USA; 2 Department of Agriculture, Nutrition, and Food Systems, University of New Hampshire, Durham, NH USA; 3 Department of Biological Sciences, North Carolina State University, Raleigh, NC USA; 4 North Carolina State University, Pamlico Aquaculture Field Laboratory, Aurora, NC USA; 5 Prestage Department of Poultry Science, North Carolina State University, Raleigh, NC USA

**Keywords:** Machine learning, Genome, Growth, Fish, Striped bass, Metabolomics, Transcriptomics

## Abstract

**Background:**

Understanding growth regulatory pathways is important in aquaculture, fisheries, and vertebrate physiology generally. Machine learning pattern recognition and sensitivity analysis were employed to examine metabolomic small molecule profiles and transcriptomic gene expression data generated from liver and white skeletal muscle of hybrid striped bass (white bass *Morone chrysops* x striped bass *M. saxatilis*) representative of the top and bottom 10 % by body size of a production cohort.

**Results:**

Larger fish (good-growth) had significantly greater weight, total length, hepatosomatic index, and specific growth rate compared to smaller fish (poor-growth) and also had significantly more muscle fibers of smaller diameter (≤ 20 µm diameter), indicating active hyperplasia. Differences in metabolomic pathways included enhanced energetics (glycolysis, citric acid cycle) and amino acid metabolism in good-growth fish, and enhanced stress, muscle inflammation (cortisol, eicosanoids) and dysfunctional liver cholesterol metabolism in poor-growth fish. The majority of gene transcripts identified as differentially expressed between groups were down-regulated in good-growth fish. Several molecules associated with important growth-regulatory pathways were up-regulated in muscle of fish that grew poorly: growth factors including *agt* and *agtr2* (angiotensins), nicotinic acid (which stimulates growth hormone production), *gadd45b, rgl1, zfp36*, *cebpb,* and *hmgb1*; insulin-like growth factor signaling (*igfbp1* and *igf1*); cytokine signaling (*socs3*, *cxcr4*); cell signaling (*rgs13*, *rundc3a*), and differentiation (*rhou, mmp17, cd22, msi1*); mitochondrial uncoupling proteins (*ucp3*, *ucp2*); and regulators of lipid metabolism (*apoa1*, *ldlr*). Growth factors *pttg1*, *egfr*, *myc*, *notch1*, and *sirt1* were notably up-regulated in muscle of good-growing fish.

**Conclusion:**

A combinatorial pathway analysis using metabolomic and transcriptomic data collectively suggested promotion of cell signaling, proliferation, and differentiation in muscle of good-growth fish, whereas muscle inflammation and apoptosis was observed in poor-growth fish, along with elevated cortisol (an anti-inflammatory hormone), perhaps related to muscle wasting, hypertrophy, and inferior growth. These findings provide important biomarkers and mechanisms by which growth is regulated in fishes and other vertebrates as well.

**Supplementary Information:**

The online version contains supplementary material available at 10.1186/s12864-024-10325-y.

## Background

The primary consumable product of a fish is the fillet, composed predominantly of fast-twitch, white skeletal muscle fibers [1]. White muscle accounts for half of the body mass of fishes [2, 3] and therefore understanding the regulatory mechanisms underlying growth of this tissue is critical for improving seafood production yields. In general, growth in vertebrates is constructed around the theory that protein synthesis is the direct fuel for somatic growth (i.e., increase in size and mass) of the animal [4]. Muscle growth in fishes differs from that of most other vertebrates in that it is indeterminate, and the majority of somatic growth is invested in the muscle tissue mass [2, 3, 5–8]. Several studies have suggested that variation in number and size distribution of muscle fibers is an important determinant of not only growth performance, but also of the textural characteristics and quality of the fillet [9, 10]. Relationships between white muscle fiber growth and whole-body growth capacity of various fish species have been reported with the intent of understanding the ability of various teleost fishes to grow rapidly and reach a comparatively large ultimate body size through the recruitment of additional white muscle fibers [2, 5, 11, 12]. While the majority of fishes show rapid somatic growth rates synchronized with dynamic increase in number of white muscle fibers (i.e., hyperplasia), a few revealed less progressive somatic growth rate synchronized with an increase in white muscle fiber diameter (i.e., hypertrophy). This suggests that hyperplasia plays a major role in fish muscle growth at early ages, wherein the longer a species can recruit new white muscle fibers, the faster is its growth rate and the larger its ultimate body size [1, 11, 13].

At the cellular level, muscle fiber growth occurs by a process called mosaic hyperplasia, in which myogenic progenitor cells (i.e., satellite cells) fuse to form new myotubes on the surface of existing muscle fibers giving rise to a mosaic of fibers [14, 15]. Satellite cells maintain muscle growth by proliferating to produce new cells or by differentiating into muscle fibers as necessary for growth [16–19]. In most fishes, mosaic hyperplasia in muscle continues until the fish reaches around 40 % of its maximum body length and often this is coincident with sexual maturity [14, 15]. Muscle growth at this point onward typically involves hypertrophy, or volumetric enlargement of the existing mosaic of muscle fibers, and this typically occurs when the specific growth rate begins to decline. The regulation of this process in fishes is complex and not well understood. It involves many different regulatory genes and enzymes involved in metabolic processes such as energy production, and structural components of the muscle itself [5].

Striped bass (*Morone saxatilis*) is an anadromous fish that typically lives most of its life in the Atlantic Ocean or estuarine tributaries and migrates into freshwater rivers to spawn. These fish have long been utilized for food and are a popular sportfish [20, 21]. The decline of the wild striped bass fishery in the mid-1980s created an opportunity to develop an aquaculture industry that primarily produced a hybrid cross of striped bass with the freshwater white bass*, Morone chrysops* [20, 22, 23]. Hybrid striped bass exhibit the benefits of hybrid vigor (heterosis) with improved survival, growth, and resistance to stress and diseases [24, 25]. Thus, hybrid striped bass gained widespread acceptance both in aquaculture for food production and as a sportfish for stock enhancement [23, 24, 26].

Presently, the molecular mechanisms of white muscle growth in hybrid striped bass, including the importance of small molecules such as metabolites and gene transcripts involved in the muscling process, are entirely unknown. Therefore, a characterization of the processes that underlie hybrid striped bass muscle growth is needed to expand understanding and potential application of findings to fisheries and aquaculture production of these and other fishes.

Here, we utilize a powerful combinatorial approach incorporating metabolomic and transcriptomic analyses to characterize the molecular mechanisms that govern growths in hybrid striped bass displaying good and poor growth under aquaculture conditions. Metabolomics is a technology that provides a global characterization and quantification of all metabolites in a biological sample [27, 28]. Metabolites are small molecules (typically less than 1 kDa in size) representative of all the components involved in the collective chemical reactions that occur within and outside of cells in an organism [29]. As metabolites underlie the entire metabolism or physiology of an organism, they represent the end point of all gene and protein activities. A series of tandem mass spectrometry (MS/MS) approaches were conducted to resolve and quantify metabolites present in the muscle and liver, as these tissues are of primary interest to somatic growth and the coordination of metabolism, respectively, in fish [30, 31]. Also, massively parallel RNA transcript sequencing (RNA-Seq; [32]) was conducted to evaluate gene expression differences in muscle tissue between fish exhibiting good and poor growth traits. As gene expression is the process by which information encoded in a gene leads to the production of an RNA transcript that may or may not lead to eventual translation into a functional protein, the metabolomics evaluation will be a strong compliment to this analysis.

The described approach also includes novel machine learning pattern recognition analyses of gene transcript expression and metabolite levels in the tissues through orthogonal and inferential statistics. Finally, global Ingenuity Pathway Analysis (IPA) was conducted to identify Canonical Pathways, Upstream Regulators, and Diseases and Functions that are not only enriched in the gene expression and metabolite datasets, but that are also predictive of fish phenotype (i.e., growth performance).

## Results

### Evaluations of growth morphometrics

**Fig. 1:**
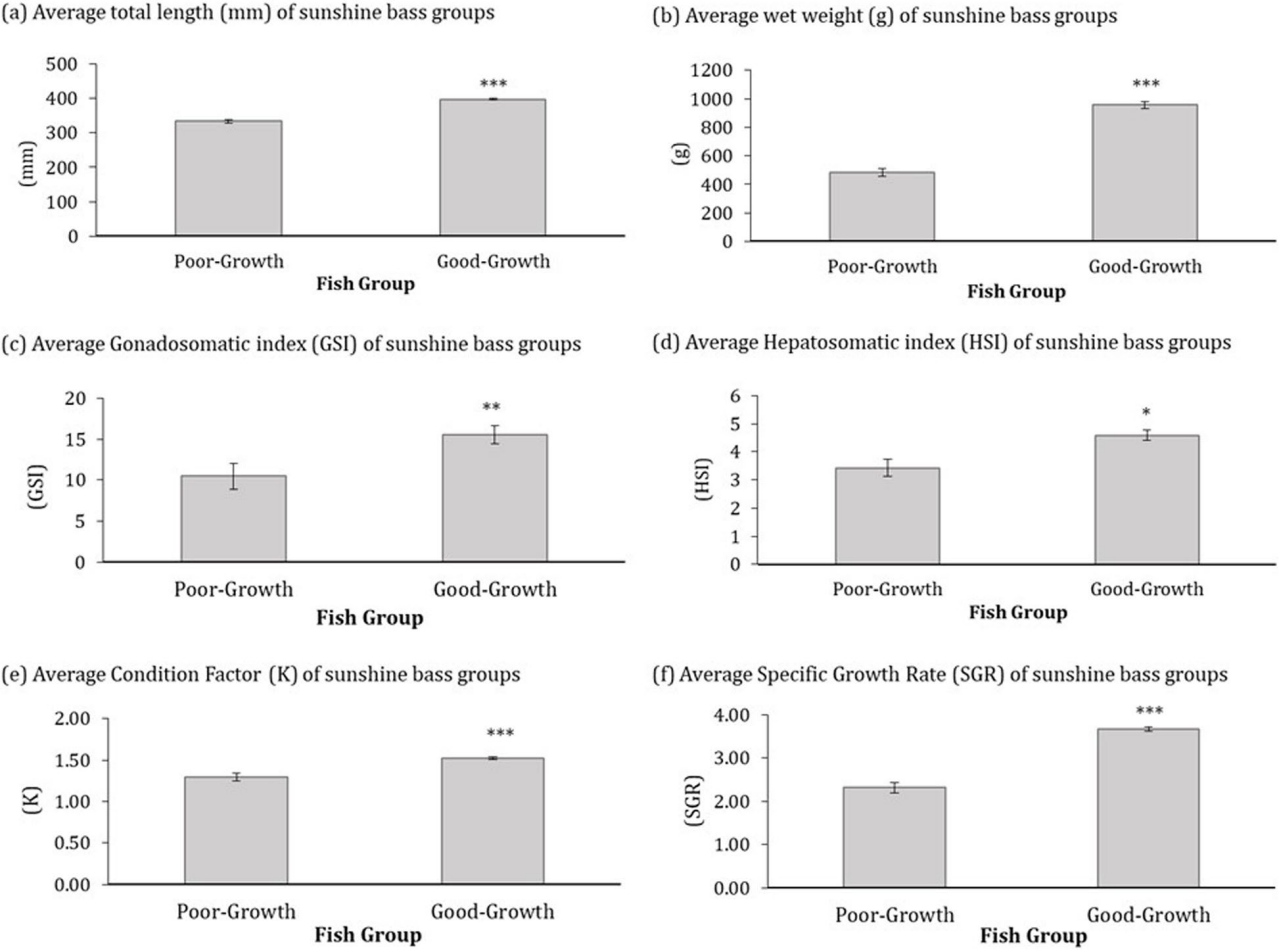
Average (**a**) total length (mm), (**b**) wet weight (g), (**c**) gonadosomatic index (GSI), (**d**) hepatosomatic index (HSI), (**e**) Fulton’s condition factor (K), and (**f**) specific growth rate (SGR) of hybrid striped bass of poor- and good-growth. Fish in each group were representative of the bottom and top 10 % in growth phenotype, respectively, among the entire production cohort. \ (Mean +/- SEM; *N* = 10) Asterisks denote significant differences between groups (*** *p* = 0.0001 ** *p* = 0.0037; * *p* = 0.0147)

The weight frequency distribution of all fish in the rearing cohort is provided in Additional file 1. Fish from the poor- and good-growth groups significantly differed in total length (*p* = 0.0001), wet weight (*p* = 0.0001), GSI (*p* = 0.0147), HSI (*p* = 0.0037), Fulton’s Condition Factor K (*p* = 0.0001), and SGR (*p* = 0.0001) (Fig. 1).

### Muscle Histology Analysis

**Fig. 2:**
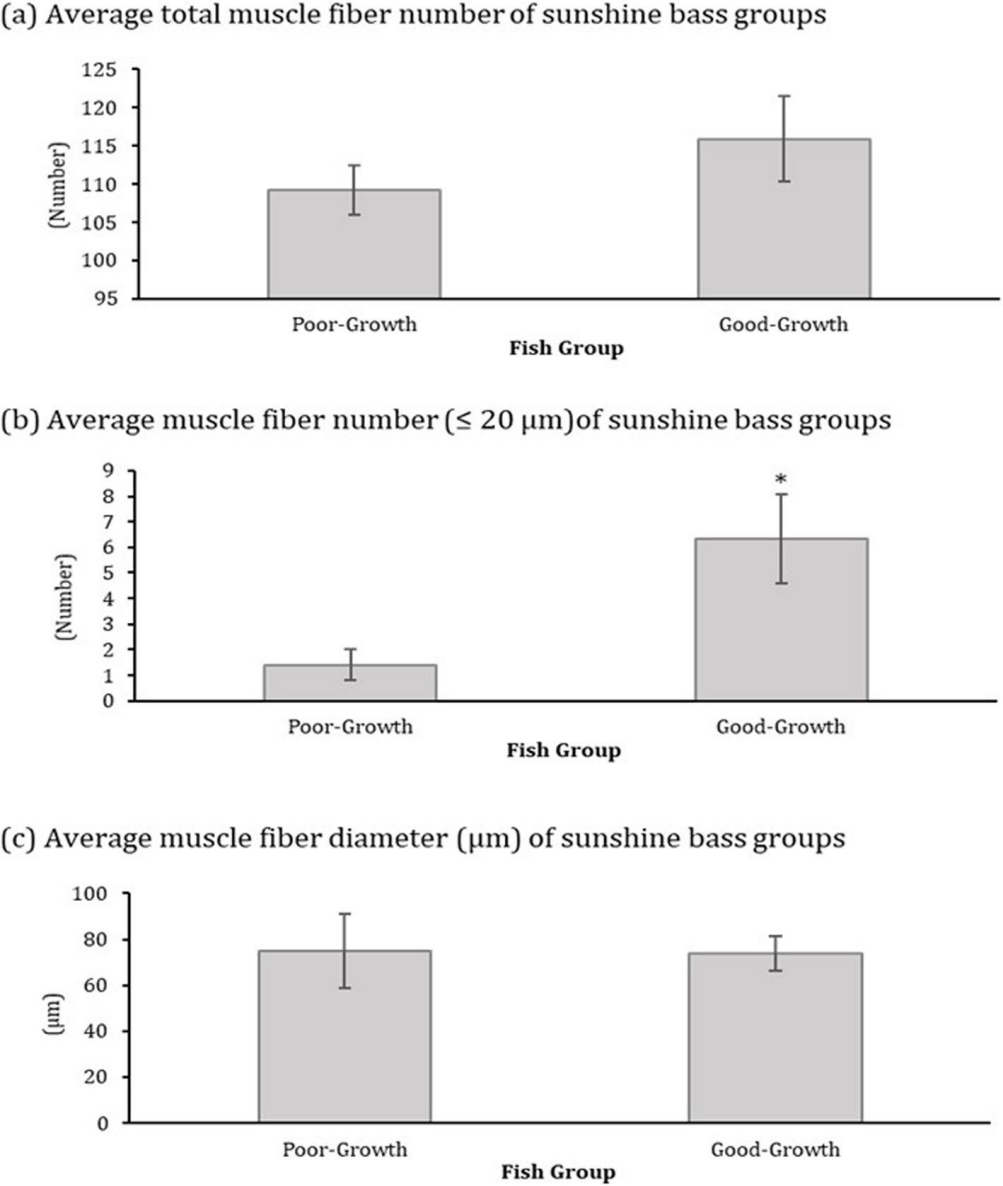
Average muscle (**a**) fiber number, (**b) **muscle fiber number ≤ 20 µm, and (**c**) muscle fiber number of hybrid striped bass in each growth group (poor-growth or good-growth, (mean +/- SEM; *N* = 5) . Asterisks denote significant differences between groups (* *p* = 0.0274)

Larger fish had both smaller and more numerous muscle fibers than smaller fish from both the poor- and good-growth groups. Fish in the good-growth group had more numerous muscle fibers that were ≤ 20 µm in diameter than those from the poor-growth group (Fig. 2, *p*=0.0274). There were no significant differences in average total muscle fiber number or in average muscle fiber diameter between growth groups. A linear regression between average total number of muscle fibers and fish wet weight of the good- and poor-growth group fish showed little correlation (R^2^=0.0858 and 0.0185, respectively) (Additional file 2) and the regression between average muscle fiber diameter and fish wet weight of the good- and poor-growth group fish showed a weak correlation (R^2^=0.1813 and 0.2165, respectively).

### Liver and muscle metabolomics analysis

A total of 469 and 464 metabolites were identified in hybrid striped bass muscle and liver tissue samples, respectively. Additional files 3 and 4 contain all data associated with these metabolites as well as the outcomes of the Welch’s Two-sample t-tests. In muscle tissue, lipids made up the greatest percentage of those identified metabolites (46.67 %). Other represented super-pathways were amino acids (20.0 %), carbohydrates, energy, nucleotides, and xenobiotics (6.67 %), and cofactors and vitamins, and peptides (3.33 %). In liver tissue, lipids made up the greatest percentage of those metabolites identified (30.0 %), followed by amino acids (20.0 %), carbohydrates and energy (13.3 %), cofactors and vitamins, nucleotides, and peptides (13.3 %), and finally, xenobiotics (3.3 %). The Principal Component Analysis (PCA) for liver tissue metabolites showed that the data tended to separate fish by growth group (Additional file 5), however separation by growth group was not as clear when examining muscle metabolites (Additional file 6); overall, PCA1 and PCA2 explained 46 % of the variance in growth.

**Fig. 3:**
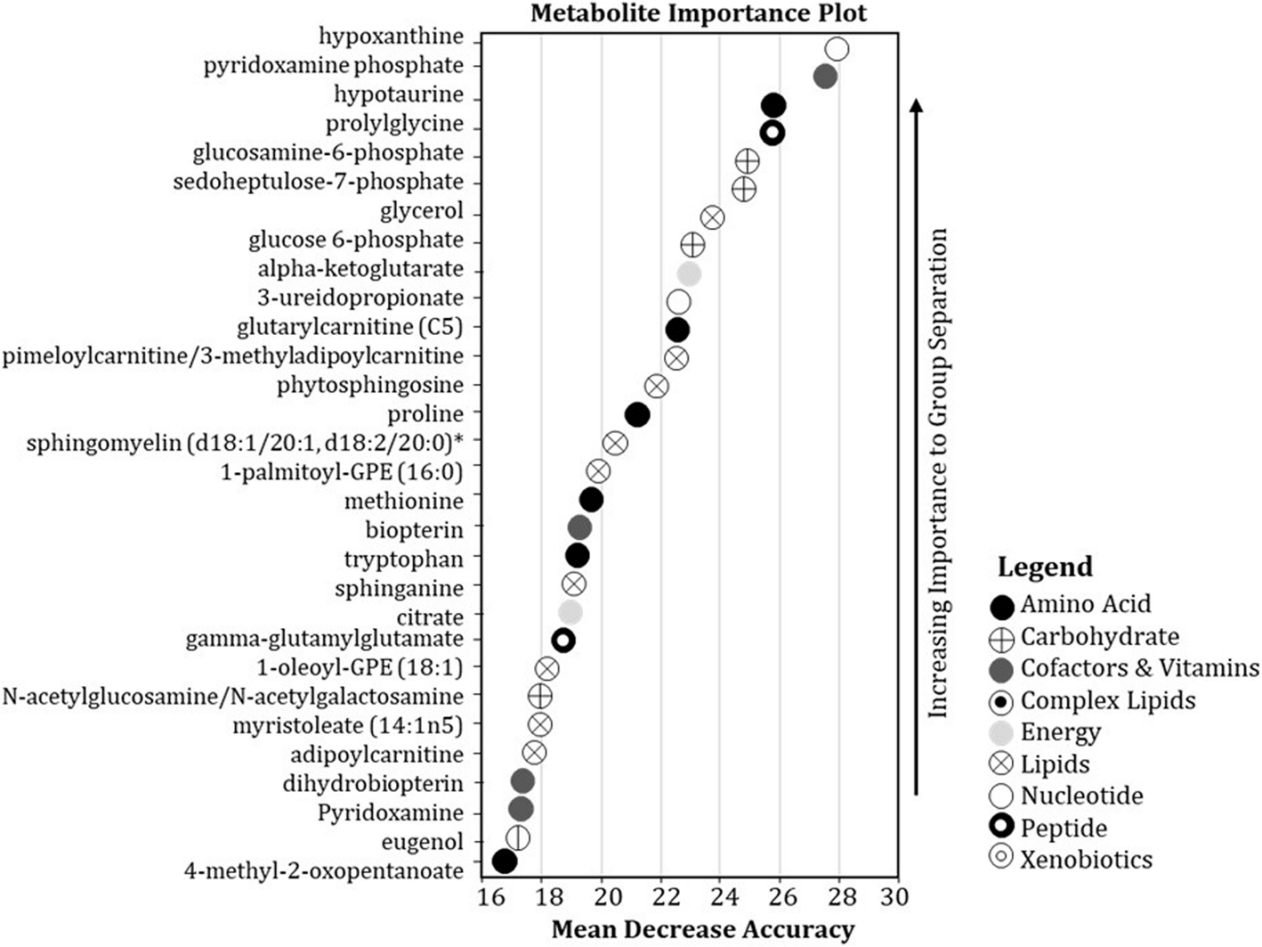
Random forest sensitivity analysis identifying the top thirty most important metabolites when comparing liver tissue of hybrid striped bass from the poor- and good-growth groups. Metabolites were ranked by increasing importance and Mean Decrease Accuracy (MDA) during random forest decision tree analysis. The legend describes the metabolic super-pathway to which each listed metabolite belongs

**Fig. 4:**
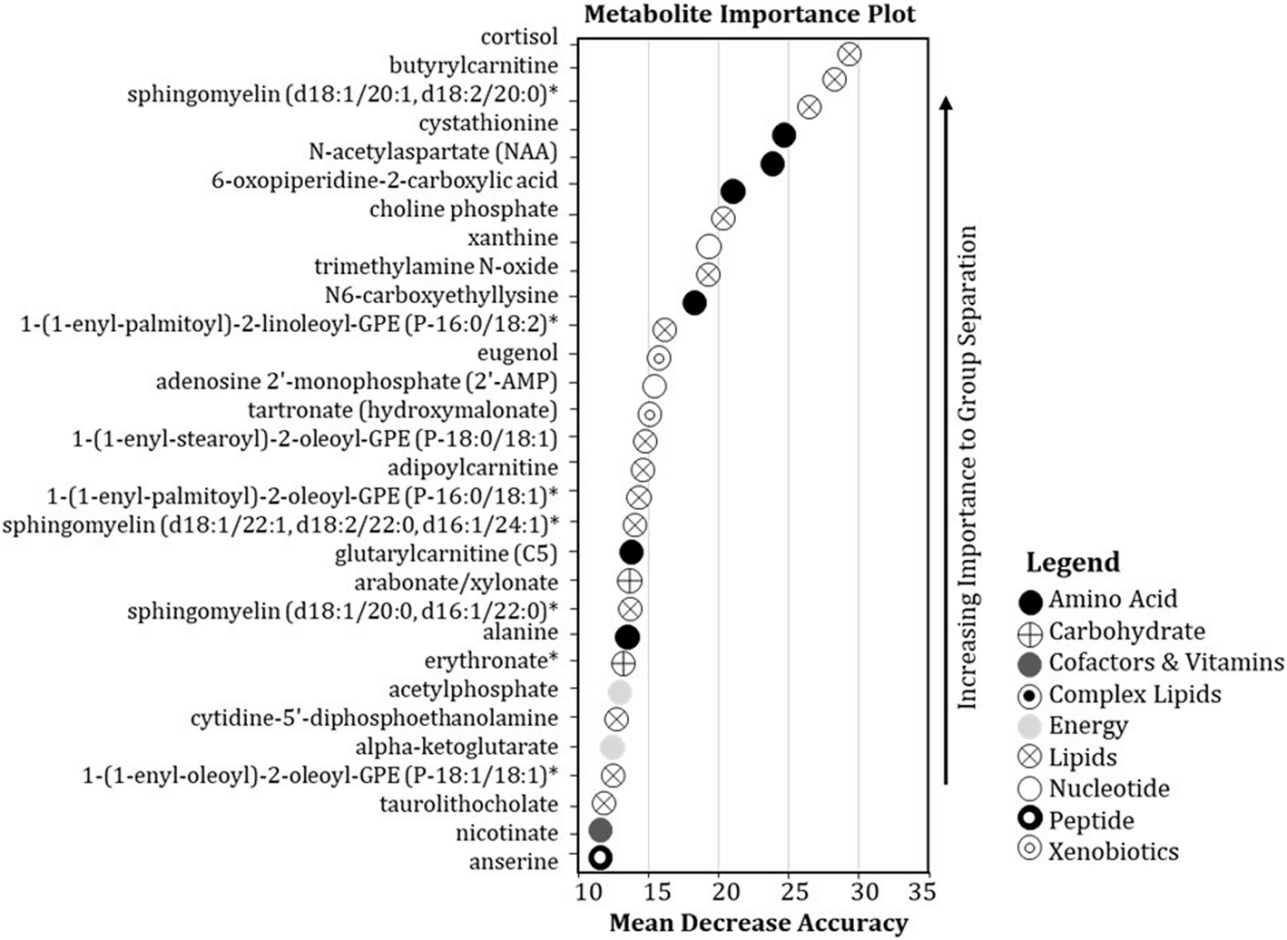
Random forest sensitivity analysis identifying the top thirty most important metabolites when comparing muscle tissue of hybrid striped bass from the poor- and good-growth groups. Metabolites were ranked by increasing importance and Mean Decrease Accuracy (MDA) during random forest decision tree analysis. The legend describes the metabolic super-pathway to which each listed metabolite belongs

The random forest machine learning analysis was effective at classifying metabolomic profiles generated from liver and muscle samples to the correct growth groups with a predictive accuracy of 89.0 % and 72.0 %, respectively. The random forest MDA sensitivity analysis identified the top thirty most important metabolites in both liver (Fig. 3) and muscle (Fig. 4) used for the classification of fish between growth groups and these metabolites were designated as meriting further investigation (i.e., akin to “statistically” significant). The super-pathways to which each of these metabolites belong are provided in Figs. 3 and 4.

### Muscle gene expression analysis

A total of 223.5 million raw RNA-Seq reads were generated and less than 0.1 % of raw reads were omitted from analysis after quality trimming (99.1 % of base pairs remained). These sequence data are deposited in the NCBI (Accession: PRJNA950439). Approximately 83.8 % of the quality trimmed reads were uniquely aligned to the striped bass genome assembly (NCBI Accession: GCA_001663605.1) and 88.9 % of these were mapped to the predicted gene space. In total, 97191 genes were identified and measured for expression. Of these, 72893 genes were informative and had annotations. The results of the PCA test conducted using the top 500 genes with highest variance among all samples showed that fish from the poor- and good-growth groups generally had similar gene expression profiles within the groups, and PCA1 and PCA2 explained 91 % of the variance in growth (Additional file 7). The numbers of expressed genes, informative genes, and the resulting differentially expressed genes identified in the comparison between the good- and poor-growth group fish are presented in Additional file 8. There were 143 muscle genes that significantly differed in expression between fish from the poor- and good- growth groups (DESeq2, *q* ≤ 0.05, FDR = 0.05); several transcript variants also were noted among these genes and likely represent the different striped bass and white bass gene copies inherited by the hybrids (Additional file 9). Among differentially expressed genes, 134 (94 %) were up-regulated in fish from the poor-growth group with an average FPKM of 21.54 and a range of 0.25 to 190.57 and only 9 (6 %) were up-regulated in fish from the good-growth group with an average FPKM of 2.63 and a range of 1.39 to 4.26 (*myog, cd300ld3, ranbp2, bicd2,* and five transcript variants of *pttg1*).

### Machine learning evaluation of muscle gene expression

All informative gene transcripts (72893) were ranked by importance *via* SVMAttributeEval and used to evaluate differences in expressed genes between good- and poor-growth groups of hybrid striped bass in a series of SMO machine learning models. The SMO overfitting models with a cross-validation of 66 % split had a percent accuracy of correctly classified instances that ranged from 66.67 % to 100 %, Kappa statistic from 0.40 to 1.00, and AUROC from 0.75 to 1.00. SMO models with cross-validation of 8-fold had an accuracy of correctly classified instances that ranged from 62.50 % to 100 %, Kappa statistic from 0.25 to 1.00, and AUROC from 0.63 to 1.00 (Additional file 10). The number of genes plotted against percentage of correctly classified instances using these approaches suggested approximately 10000 genes or less were optimal for predicting growth group of the fish (to avoid overfitting). To further reduce the number of genes to only the top, most important predictors of fish growth (to avoid underfitting), an orthogonal approach was applied, whereby the top 10000 genes ranked by SVMAttributeEval were used as baseline for another series of SMO models. The result verified that the top 1000 to 10000 genes were all important for classification (correctly classified instances 100 %, Kappa statistic 1.00, and AUROC 1.00) and that model underfitting began to occur at around 500 or fewer gene inputs (correctly classified instances < 100 %, Kappa statistic < 1.00, and AUROC < 1.00; Additional file 11). The top 150 ranked genes were chosen for pathway analysis (i.e., akin to “statistically” significant) based on underfitting decomposition of model performance when 150 or fewer gene input values were included. Negative control models for machine learning performed at approximately 50 % correct classification as predicted based on the Law of Probability, indicating that learning during training was true.

Of the 150 top important genes, 36 (24 %) were up-regulated in muscle of fish from the poor-growth group with an average FPKM of 4.00 that ranged from 0.11 to 42.89, whereas 114 genes (76 %) were up-regulated in muscle of fish from the good-growth group, with an average FPKM of 2.67 that ranged from 0.20 to 16.90 (Additional file 12).

### Combinatorial muscle pathway analysis

The two gene lists (143 genes with an inferential statistics *q*-value cutoff of differential expression at ≤ 0.05 FDR = 0.05 and 150 genes ranked by SVMAttributeEval machine learning) were compared and the shared genes identified from both ranking procedures are reported in Additional files 9 and 12. Eleven genes, including their transcript variants, were shared between the two ranking approaches and they were all up-regulated in muscle of fish from the poor-growth group (*agtr2, cd22, cebpb, cxcr4, gadd45b, mmp17, msi1, rgl1, rgs13, rhou,* and *rundc3a*).

**Fig. 5:**
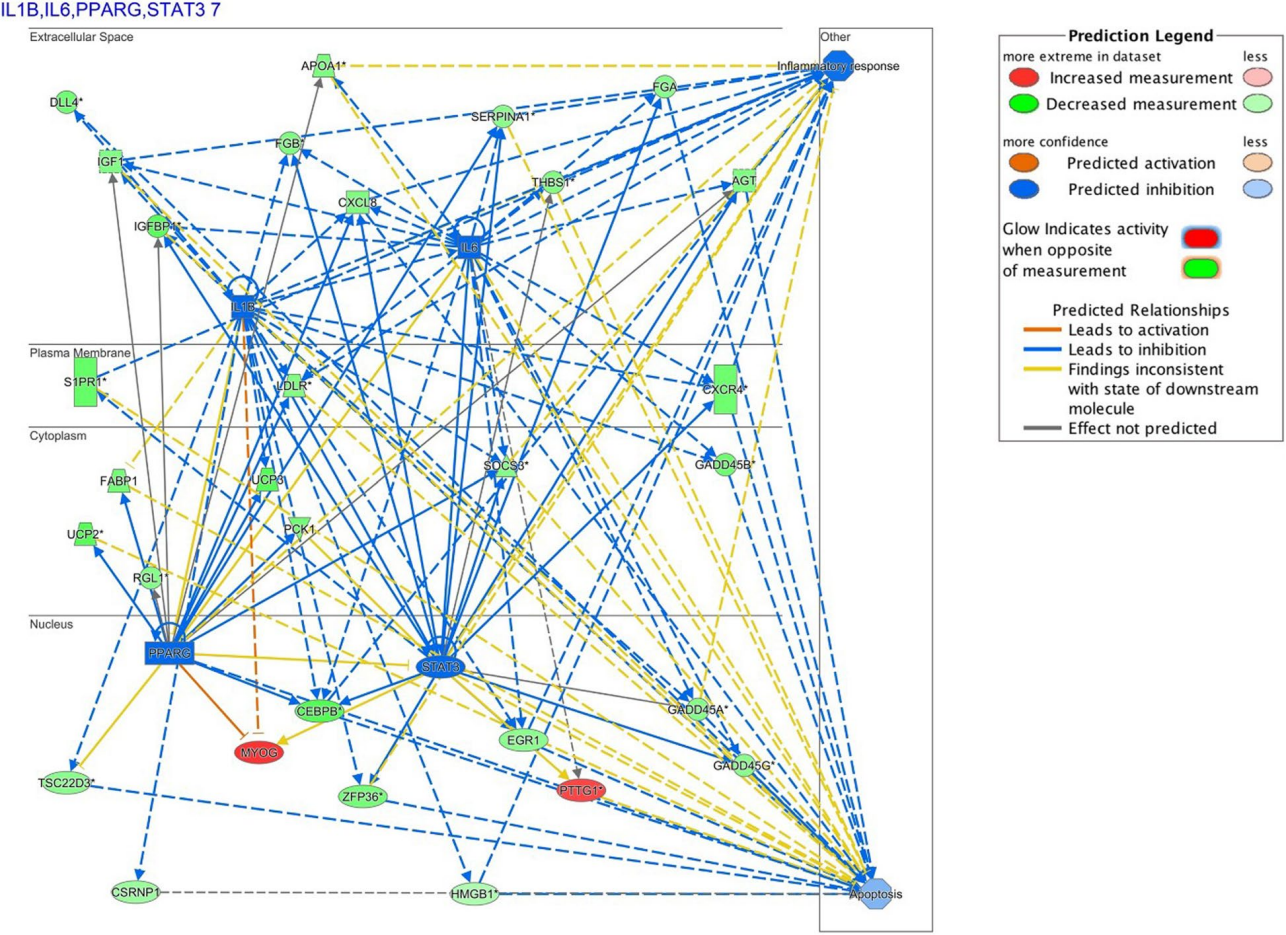
Upstream Regulator analysis based on muscle metabolites and genes identified by inferential statistics (FDR, *q* ≤ 0.05) in hybrid striped bass. Measured gene expression and metabolite values were predicted to inhibit 4 major regulatory factors (*il6, il1b, stat3, pparg*) in muscle of fish from the good-growth group. The network indicates direct relationships of inhibited factors (*blue*) leading to inhibition of inflammatory response and apoptosis in muscle of fish from the good-growth group. Down-regulation (*green*) refers to lower gene expression or metabolite levels measured in muscle of fish from the good-growth group relative to the poor-growth group, while up-regulation (*red*) refers to increased levels measured in muscle of fish from the good-growth group relative to the poor-growth group. Connections are drawn between molecules that have been found to have relationships in the literature. Arrows indicate activation and perpendicular lines indicate inhibition. Image was created using Ingenuity Pathway Analysis Upstream Regulator Analysis (Qiagen IPA, Germantown, MD, USA)

Hybrid striped bass gene transcripts and metabolites were mapped to the Qiagen Ingenuity Knowledge Base and the following validated (informative) entries were retrieved and used for pathway analysis: 142 out of 143 genes that were ranked by *q*-value of differential expression at ≤ 0.05 (inferential statistics); 145 out of 150 genes ranked by SVMAttributeEval (machine learning); and 358 out of 469 metabolites. The Fisher’s Exact Test identified the following genes and metabolites significantly enriched in the pathway analysis (*p*-value ≤ 0.05): 57 genes ranked by inferential statistics of which 53 were down-regulated and 4 up-regulated in fish from the good-growth group relative to the poor-growth group; 96 genes ranked by machine learning of which 30 were down-regulated and 66 up-regulated in fish from the good-growth group; and 39 metabolites of which 19 were down-regulated and 20 up-regulated in fish from the good-growth group. Each of the combined gene and metabolite lists included the following numbers of molecules that were significantly enriched for pathways: 70 molecules of which 56 were down-regulated and 14 up-regulated in fish from the good-growth group (genes ranked by inferential statistics and all metabolites) and 117 molecules of which 41 were down-regulated and 76 up-regulated in fish from the good-growth group (genes ranked by machine learning and all metabolites). All significantly enriched canonical pathways identified by IPA for white muscle ranked by *q*-value and SVMAttributeEval are shown in Additional files 13 and 14; pathway networks are provided in Figs. 4, 5, and 7 and Additional files 15, 16, 17, 18.

## Discussion

Hybrid striped bass in the good- and poor-growth performance groups differed significantly in average weight, total length, GSI, HSI, Fulton’s condition factor K, and SGR (Fig. 1), indicating that the fish were representative of different extremes of the size distribution and experienced different growth states observed amongst the rearing cohort at the time of sampling. Fish from the good-growth group had a significantly greater number of smaller diameter (≤ 20 µm) muscle fibers and fish from the poor-growth group had a greater proportion of larger, hypertrophic muscle fibers, however the relationship between average muscle fiber diameter and fish weight was weak (Fig. 2). Collectively, these findings suggest that fish from the good-growth group have a greater degree of muscle hyperplasia compared to the poor-growth group, which may have precociously switched to hypertrophic growth. This shift in muscle growth pattern may underlie the overall difference in fish growth performance observed in this study.

The statistical and machine learning approaches of examining data at the gene and metabolite levels that led to the combinatorial pathway analyses performed identified important biomarkers and regulators of growth performance, and provided enriched pathways and functions that are predictive of fish growth phenotype based on the observed data.

### Inflammation, cholesterol metabolism, and cortisol

The most significant pathways identified from the list of muscle metabolites and genes identified by inferential statistics were acute phase response signaling and LXR/RXR activation (Additional file 13). Inhibition of these two pathways in muscle was predicted for fish from the good-growth group. The acute phase response signaling pathway provides a rapid, non-specific inflammatory defense in the organism [33–35], while LXR/RXR is involved in regulation of lipid metabolism, inflammation, and cholesterol to bile acid catabolism [36–38]. Cholesterol levels in liver were not different between fish, however cholate and taurocholate showed elevations in both the liver (Additional file 19) and muscle (Additional file 20) of fish from the poor-growth group, albeit this response was statistically insignificant. The anti-inflammatory hormone cortisol was significantly elevated in muscle of fish from the poor-growth group, and it also was the most important muscle metabolite predictor of growth performance identified by random forest MDA (Fig. 4). The hormone is also a well-established “stress” factor that induces catabolism of carbohydrate, protein and lipid stores in vertebrates, including fishes.

**Fig. 6:**
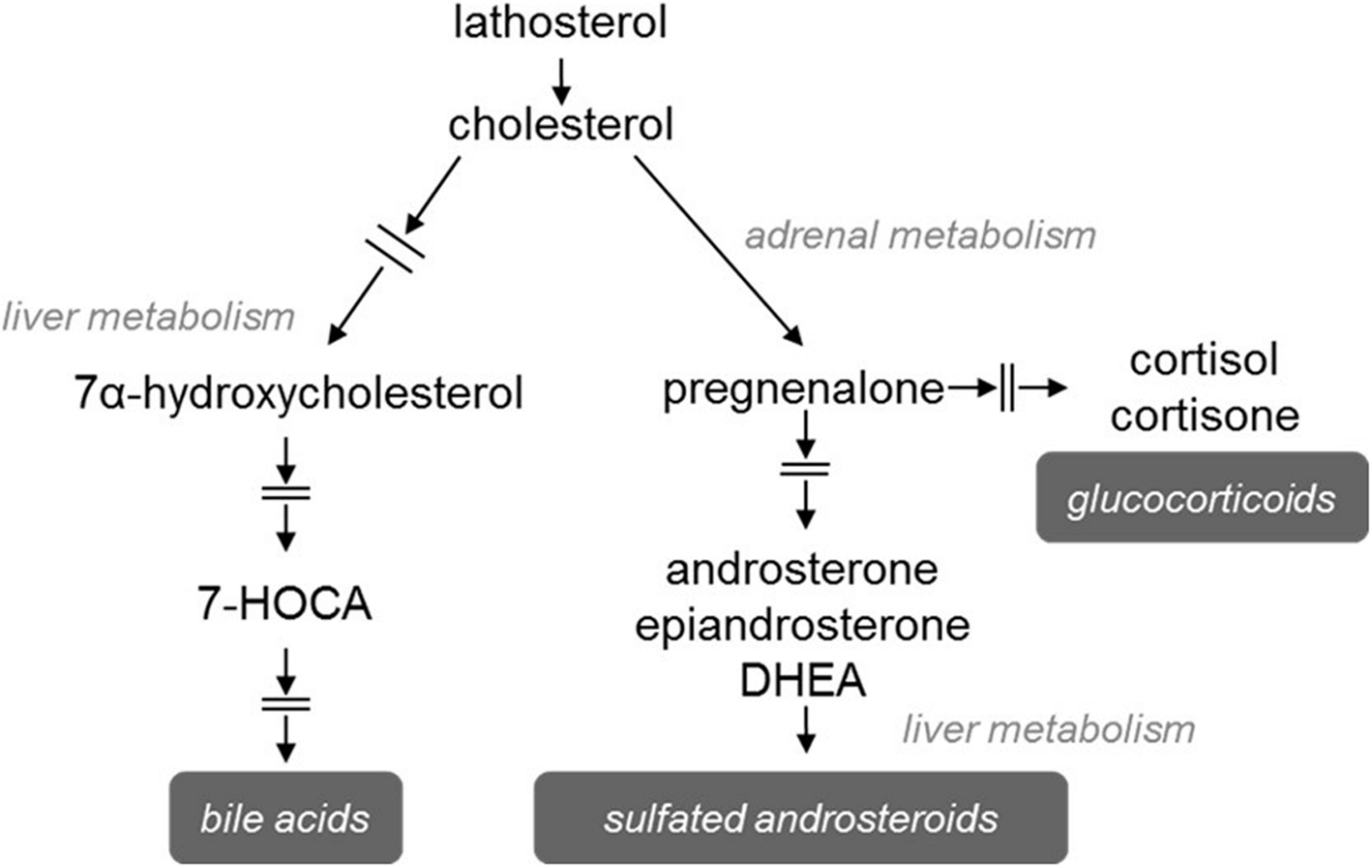
Diagram of the cholesterol metabolic pathway that includes cortisol and bile acid by-products. The pathway shown corresponds to several liver metabolites and muscle gene regulatory pathways identified as important for growth in hybrid striped bass; metabolites were typically elevated in fish from the poor-growth group

A common set of upstream regulators predicted to be inhibited in muscle of fish from the good-growth group were cytokines and other regulators related to inflammation including *stat3, pparg, il1b,* and *il6* [39–46] (Fig. 5). Significantly lower levels of the pro-inflammatory eicosanoid 12-HETE (along with a non-significant decreases in 15-HETE and 12-HEPE) in muscle of fish from the good-growth group may suggest reduced inflammation, which would positively affect growth (Additional file 20) [47–49]. This indicates overall that elevated cortisol levels in poor-growth group fish may be associated with dysfunctional inflammation or cholesterol or steroid metabolism that led to the observed growth differences (Fig. 6). For example, an inability to effectively clear cortisol from the blood following a stress event, which is why it may remain elevated in fish from the poor-growth group. Conversely, cortisol may be produced in fish from the poor-growth group in response to localized muscle inflammation. In either case, cortisol is typically cleared from the blood by the liver and persistent inflammation and elevated cortisol levels lead to muscle wasting, poor muscle growth, and decreased feeding behavior [50–53]. Inflammation of liver was identified as activated in fish from the good-growth group, however, the importance of this observation is presently unclear. Further studies assessing plasma cholesterol levels may identify whether these poor-growth group fish might have been exposed to greater rearing stress, experience low cholesterol demand, or suffer from some form of liver dysfunction that may underlie poor muscle growth in addition to the evaluations presented here. Additionally, nutritional status also may be a factor that influences inflammation [54]. Omega-3 and omega-6 polyunsaturated fatty acids (PUFAs) may be utilized to produce anti-inflammatory metabolites [55]. These compounds may possibly improve growth and reduce inflammation if included in the diet of hybrid striped bass raised in aquaculture.

### Cell senescence, proliferation, and apoptosis

The cellular senescence pathway also was predicted to be significantly inhibited in muscle of fish from the good-growth group based on the list of metabolites and genes identified by inferential statistics (Additional file 13). In particular, expression of *cebpbeta, cxcl8*, *gadd45a, gadd45b*, and *gadd45g* were all down-regulated. This is further predicted to lead to increased proliferation of cells [56, 57] and reduced cellular senescence [58], which may underlie, in part, the greater hyperplastic growth of muscle cells in fish from the good-growth group (Additional file 15). By comparison, activation of the senescence pathways in fish from the poor-growth group may have led to the precocious switch from hyperplasia to hypertrophy as observed by muscle histology (Fig. 2). Furthermore, apoptosis and necrosis were predicted to be inhibited in muscle of fish from the good-growth group, indicating possibly lower cell death rate and hence superior muscle growth phenotype (Fig. 5). The network represented in Additional file 16 indicates that proliferation of myofibroblasts was predicted to be activated in muscle of fish from the good-growth group, but many other functions related to growth or proliferation of muscle cells were generally inhibited. Therefore, some of these identified pathways may support or contrast the phenotype observed in fish from the good-growth group. Significantly elevated choline phosphate and glycerophosphoethanolamine (GPE) levels and non-significantly elevated levels of glycerophosphorylcholine (GPC) in muscle of fish from the good-growth group further support active cell growth and suggest increased phospholipid turnover or plasma membrane remodeling (Additional file 21), which would be expected during active cell hyperplasia as observed.

### Cell receptor signaling and nucleotide and amino acid synthesis

**Fig. 7:**
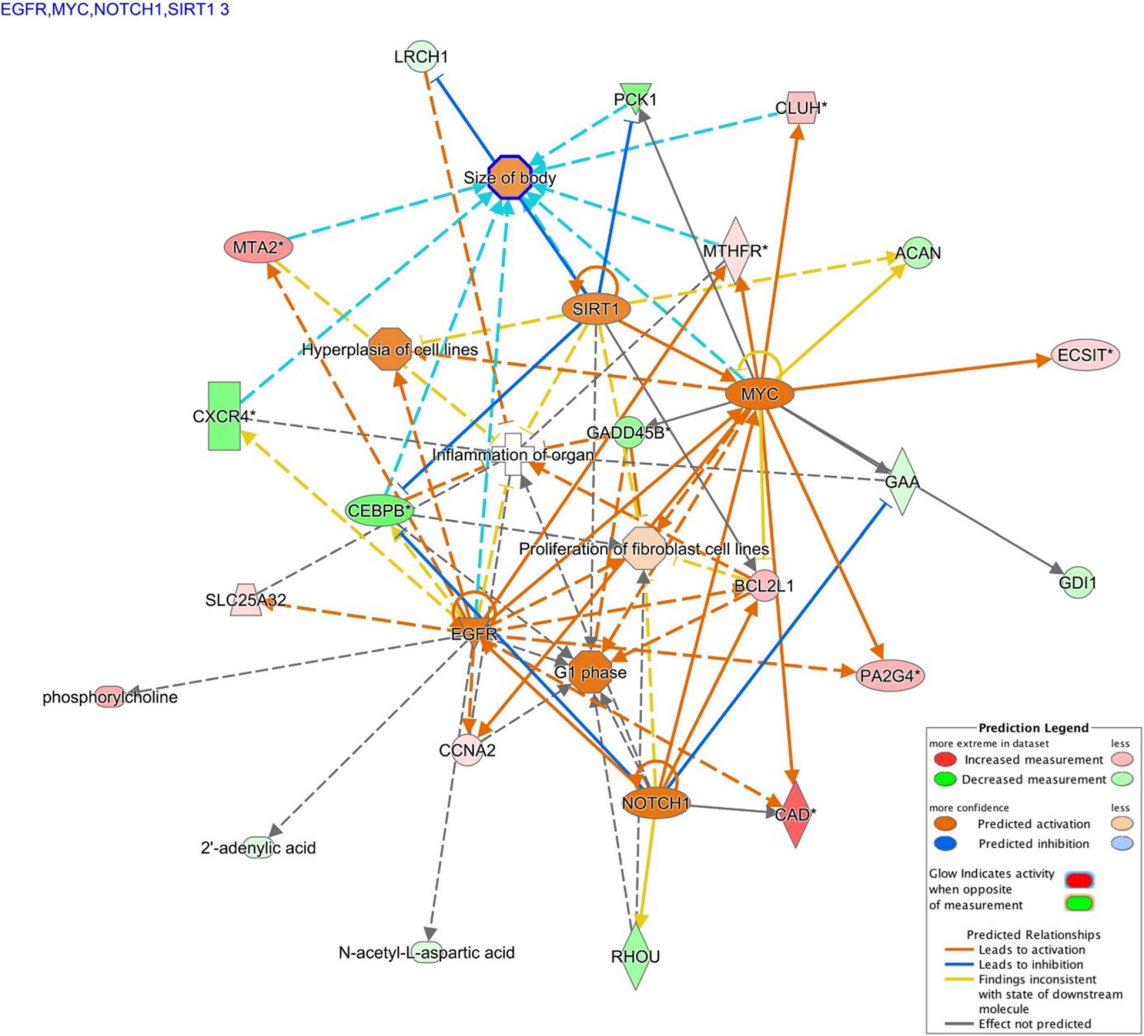
Upstream Regulator analysis based on metabolites and genes identified by machine learning (SVMAttributeEval). Measured gene expression and metabolite values were predicted to activate 4 major regulatory factors (*myc, sirt1, egfr, notch1*, in blue text top-left) in muscle of fish from the good-growth group. The network indicates direct relationships of activated factors (*orange*) leading to activation of hyperplasia of cell lines, proliferation of fibroblasts, size of body, and G1 phase (cell growth) in muscle of fish from the good-growth group; blue lines indicate inhibition effects. Down-regulation (*green*) refers to lower gene expression or metabolite levels measured in muscle of fish from the good-growth group relative to the poor-growth group, while up-regulation (*red*) refers to increased levels measured in muscle of fish from the good-growth group relative to the poor-growth group. Connections are drawn between molecules that have been found to have relationships in the literature. Arrows indicate activation and perpendicular lines indicate inhibition. Image was created using Ingenuity Pathway Analysis Upstream Regulator Analysis (Qiagen IPA, Germantown, MD, USA)

The most clearly defined pathway identified from the list of muscle metabolites and genes ranked by machine learning was B cell receptor signaling (Additional file 14). When IPA *Molecule Activity Predictor* was used for the pathway, it was shown that up-regulation of *bcl-x* and *creb* and down-regulation of *cd22* predictively leads to activation of gene transcription and inhibition of apoptosis (Additional file 17). B cell activation is crucial for cellular development and is involved in the activation of many downstream pathways, from inhibition of apoptosis to accumulation of muscle protein [59, 60]. These results show an increase in activation of B cell receptor signaling in muscle of fish from the good-growth group, correlating positively with the observed phenotype of better muscle growth and reduced cell death. Expression of four upstream regulators were found to be significantly different (*egfr, myc, notch1*, and *sirt1*) (Fig. 7) and all have previously characterized crucial roles in development and growth [61–67]. Indeed, these genes are all up-regulated in muscle of fish from the good-growth group, thus leading to the conclusion that all of these four regulators are involved in pathway activation and the functional profile observed in fish from the good-growth group (Additional file 18). Activation of *egfr, myc, notch1* and *sirt1* are positively related to body size, cell cycle control, proliferation of fibroblasts, growth of connective tissue, and hyperplasia of cell lines [68–75]. Metabolites in the analysis show enrichment of mainly nucleotide and amino acid synthesis pathways. Overall, the data suggest activation of gene transcription in the B cell receptor signaling pathway that leads to amino acid and nucleic acid synthesis in muscle of fish from the good-growth group, supporting active anabolism and growth (Additional file 17).

Signs of significantly increased amino acid demand in liver were observed in fish from the good-growth group. Measured levels of several amino acids (e.g., aspartate, glutamine, histidine, isoleucine, leucine, methionine, proline, serine, threonine, tryptophan, tyrosine, and valine) were lower in liver, although not all were significantly so (Additional file 22). This may be expected given the high metabolic demand of hyperplastic muscle growth in fish from the good-growth group. Depletion of these amino acids in liver might be related to their secretion and incorporation into protein by growing muscle tissue and/or their direct use for production of energy to support active metabolism [76]. Significantly lower levels of urea, ornithine, and citrulline in liver of fish from the good-growth group (Additional file 23) support secretion and incorporation of these amino acids into muscle proteins in context of the observed depressed liver amino acid levels. Interestingly, creatine and creatinine were both significantly elevated in liver of fish from the good-growth group (Additional file 23), which could suggest a redirection of arginine toward creatine production to supplement high energetic demand. By contrast, the higher levels of amino acids in liver of poor-growth fish may suggest enhanced proteolysis or reduced protein synthesis which could be mediated by the catabolic actions of cortisol ultimately resulting in growth suppression.

Metabolites derived from catabolism of branched-chain amino acid (BCAA; isoleucine, leucine and valine) can enter gluconeogenesis or the citric acid cycle for energy production. Leucine and isoleucine levels were significantly lower in liver of fish from the good-growth group and, although not statistically significant, valine was also depleted (Additional file 22), suggestive of changes in amino acid availability or catabolic use. Consistent with catabolic use, the keto-acids 4-methyl-2-oxopentanoate, 3-methyl-2-oxovalerate, and 3-methyl-2-oxobutyrate were also significantly elevated in liver of fish from the good-growth group (Additional file 24). While signs of liver BCAA catabolism were observed, succinate and succinylcarnitine (a surrogate for succinyl-CoA) were not different between fish from the growth groups (Additional file 24), perhaps suggestive of declining BCAA catabolic rate in fish of the good-growth group at the time of sampling. The data, however, suggest overall that fish from the good-growth group catabolize BCAA for energy production in liver (Additional file 25) and may also use other free amino acids for protein polymerization in liver and presumably muscle as well (i.e., protein accretion). This observation would be consistent with a shift towards anabolism in the muscle, which is observed as hyperplasia in fish from the good-growth group. Interestingly, dietary supplementation with leucine promotes weight gain in rainbow trout [77], which may suggest dietary BCAA supplementation could positively affect growth in hybrid striped bass as well.

Significantly elevated levels of sedoheptulose-7-phosphate and AICA ribonucleotide (AICAR) in liver of fish from the good-growth group suggest active glucose use for purine nucleotide synthesis (Additional file 26). This is further supported by significantly lower levels of hypoxanthine and xanthine in liver from good-growth group fish, with a trend toward lower levels of allantoin as well (Additional file 26); hypoxanthine also was the most important liver metabolite predictor of growth status by random forest MDA (Fig. 3). This contention is consistent with trends toward decreases in liver adenosine 3’-monophosphate (3’-AMP) and adenosine 3’,5’-diphosphate (ADP) levels, although guanosine 5’- monophosphate (5’-GMP) was significantly elevated (Additional file 26). In muscle of fish from the good-growth group, levels of xanthine, adenosine 2’-monophosphate (2’-AMP), and guanine levels were significantly lower compared to fish from the poor-growth group; there also was a trend for muscle inosine and allantoin levels to be lower in fish from the good-growth group (Additional file 27). Collectively these findings support purine nucleotide synthesis demand in muscle of fish from the good-growth group. These nucleotides could be used as substrate for RNA or DNA synthesis in rapidly dividing cells such as those observed in the hyperplastic muscle (Fig. 2) and RNA and DNA content of tissues has been used as a reference of cell growth or proliferation in previous studies [78]. Interestingly, the nucleotide sugars UDP-N-acetylglucosamine and UDP-N-acetylgalactosamine showed a trend, although non-significant, toward elevation in muscle of fish from the poor-growth group (Additional file 28). This could suggest a shift in glucose use for hexosamine synthesis, which has been shown to function during nutrient sensing that might lead to insulin resistance in mammals [79]. Such a response might indicate some form of metabolic dysfunction or dietary insufficiency in fish from the poor-growth group.

### Liver bioenergetics

**Fig. 8:**
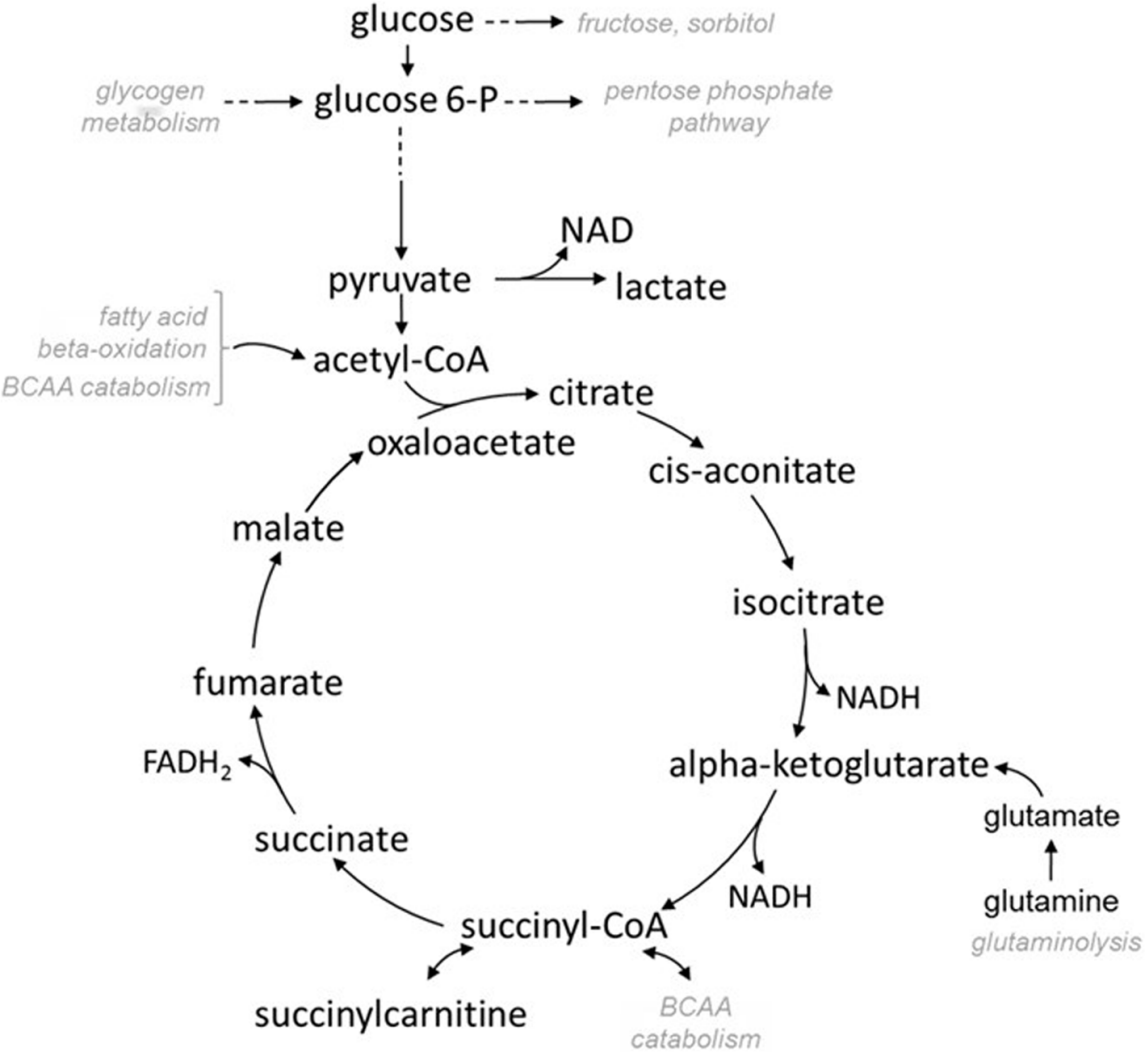
Diagram of the citric acid cycle where the grey sub-pathways shown correspond to related metabolites and genes identified as important for growth in hybrid striped bass

Carbon can flow into the citric acid cycle from a number of sources, including carbohydrates (entering as pyruvate through glycolysis), glutamine (entering as alpha-ketoglutarate), BCAA (entering as acetyl-CoA and succinyl-CoA), and lipids (entering as keto acids through beta-oxidation) via conversion of acetyl-CoA to citrate (Fig. 8). Glucose levels in liver of fish from the good-growth group were non-significantly depressed compared to those in the poor-growth group (Additional file 29). Significant increases in glucose-6-phosphate, fructose-6-phosphate, and dihydroxyacetone phosphate (DHAP) levels in liver of fish from the good-growth group (Additional file 29) suggests increased glycolytic use in the liver. In contrast, non-significant increases in phosphoenolpyruvate (PEP) and 3-phosphoglycerate levels (Additional file 29) in liver are consistent with decreased glycolytic use in fish from the poor-growth group. The glycolytic end-product pyruvate showed a trend toward increased levels in liver of fish from the good-growth group (Additional file 26), while lactate was not different, suggestive of increasing glycolytic input into the citric acid cycle. Glucose level, however, was also significantly elevated in muscle of fish from the good-growth group and pyruvate level in muscle did not differ between fish (Additional file 28). This could reflect secretion of some glucose by the liver and uptake by muscle of fish from the good-growth group to support active muscle metabolism.

Significantly elevated levels of citrate and along with a trend toward depressed levels of acetyl-CoA in liver of fish from the good-growth group may suggest increased input of acetyl-CoA into the citric acid cycle, which would be consistent with higher glycolytic use of glucose (Additional file 30). By contrast, fish from the poor-growth group had non-significantly elevated levels of liver acetyl-CoA and very low levels of citrate, indicating decreased input into the citric acid cycle (Additional file 30). The trend toward increased acetylphosphate, which is produced from the conversion of pyruvate to acetyl-CoA, in fish from the good-growth group further suggests increased glycolytic input into the citric acid cycle (Additional file 30). Similarly, significantly elevated levels of alpha-ketoglutarate in fish of the good-growth group could indicate increased glutaminolysis, as glutamine was also significantly lower in liver of fish from the good-growth group (Additional file 30), along with several other amino acids discussed above (Additional file 22). Overall, the findings support active energy production via glycolysis and the citric acid cycle among fish from the good-growth group, which indicates an active metabolic growth state. A non-significant trend toward elevated maltohexaose, maltopentaose, maltotetraose in liver of fish from the good-growth group (Additional file 31) could suggest glycogen breakdown with the resulting glucose available for use in glycolysis/citric acid cycle or for purine nucleotide synthesis as discussed above. Muscle metabolite levels collectively suggest that fish from the good-growth group primarily metabolize glucose into pyruvate for citric acid cycle or lactate for gluconeogenesis.

Energetics pathways appeared to be impaired in fish from the poor-growth group, particularly in regard to lipid metabolism. Fatty acid omega-oxidation may serve as a rescue pathway when beta-oxidation is impaired and it may also supplement beta-oxidation at times of extreme oxidative demand [80, 81]. While dicarboxylate fatty acids (DFAs) themselves were below the threshold of detection in both liver and muscle tissues, differences in DFA-carnitine conjugate levels suggest a subtle shift in fatty acid oxidative efficiency of fish from the poor-growth group compared to the good-growth group. Significant decreases in adipoylcarnitine and pimeloylcarnitine/3-methyladipoylcarnitine levels with a non-significant trend for decreased carnitine-dicarboxylate fatty acid conjugate levels in liver of good-growth group fish suggests a reduced fatty acid omega-oxidation rate (Additional file 32). Adipoylcarnitine also showed a non-significant decrease in muscle of fish from good-growth group (Additional file 33). Collectively, low levels of omega- or beta-oxidation compounds in the tissues of good-growth group fish suggest that they are not stressed and are growing well. Conversely, elevated levels of these compounds in fish from the poor-growth group suggest metabolic dysfunction, exposure to some form of stress as indicated by increased cortisol, or dietary insufficiency leading to fatty acid beta-oxidation.

### Thiamin (Vitamin B) deficiency

While thiamin (Vitamin B1) was below threshold of detection in muscle and liver, thiamin diphosphate (also known as thiamin pyrophosphate), showed non-significant elevation in muscle of fish from the good-growth compared to the poor-growth group. The biological function of thiamin pyrophosphate, which is the active form of thiamin, is to serve as a cofactor for several enzymes important for biosynthesis of many cell constituents, neurotransmitters, pentoses as nucleic acid precursors, and for production of reducing equivalents used in oxidant stress defense. It also functions as a coenzyme in carbohydrate metabolism, making keto analogues from amino and fatty acid metabolism available for production of energy [82]. Thiamin pyrophosphate on the pyruvate dehydrogenase enzyme binds to pyruvate and thus plays an important role in glucose homeostasis in muscle tissues. Activation of the complex promotes catabolism of glucose, whereas inactivation conserves substrates for hepatic gluconeogenesis [83]. Decreased levels of pyruvate and elevated levels of thiamin pyrophosphate in muscle of fish from the good-growth group, with concurrent lower levels of liver glucose and significantly elevated levels of muscle glucose in fish from the good-growth group suggest activation of pyruvate dehydrogenase enzyme, which promotes disposal of glucose from the liver to the blood (i.e., muscle). In contrast, decreased levels of thiamin pyrophosphate in muscle of fish from the poor-growth group with elevated levels of pyruvate and increased levels of liver glucose, parallel with a significant decrease in muscle glucose suggests some sort of metabolic deficiency, where pyruvate is not being catalyzed. Overall, this suggests thiamin deficiency or dysfunction of the pyruvate dehydrogenase complex in fish of the poor-growth group.

The cause of potential thiamin deficiency in fish from the poor-growth group remains unclear, however, it could be that thiamin is heat denatured during the feed extrusion process or that poor-growth fish perhaps do not get access to enough feed, and hence have marginal thiamin intake. To make up for food deprivation, these fish may have foraged on crustaceans, small prey fish, or other live food sources during the pond stage of rearing, all of which may contain thiaminases that degrade dietary thiamin included in the prepared feed. Studies have shown that thiaminases are present in the viscera of fish species, including white bass [84]. From a genetic point of view, it could be that poor-growth hybrid striped bass lack the gene(s) responsible for mitigating thiaminase metabolism, which might also lead to poor-growth performance. Animals suffering from thiamin deficiency due to thiaminases generally respond if administered thiamin and/or by removing the source of thiaminase from the diet. However, since thiaminase metabolism could have been inherited, future studies should target genes that are responsible for activating or inhibiting metabolomic activity of thiaminases (e.g., *thi20*, hydroxymethylpyrimidine kinase).

**Fig. 9:**
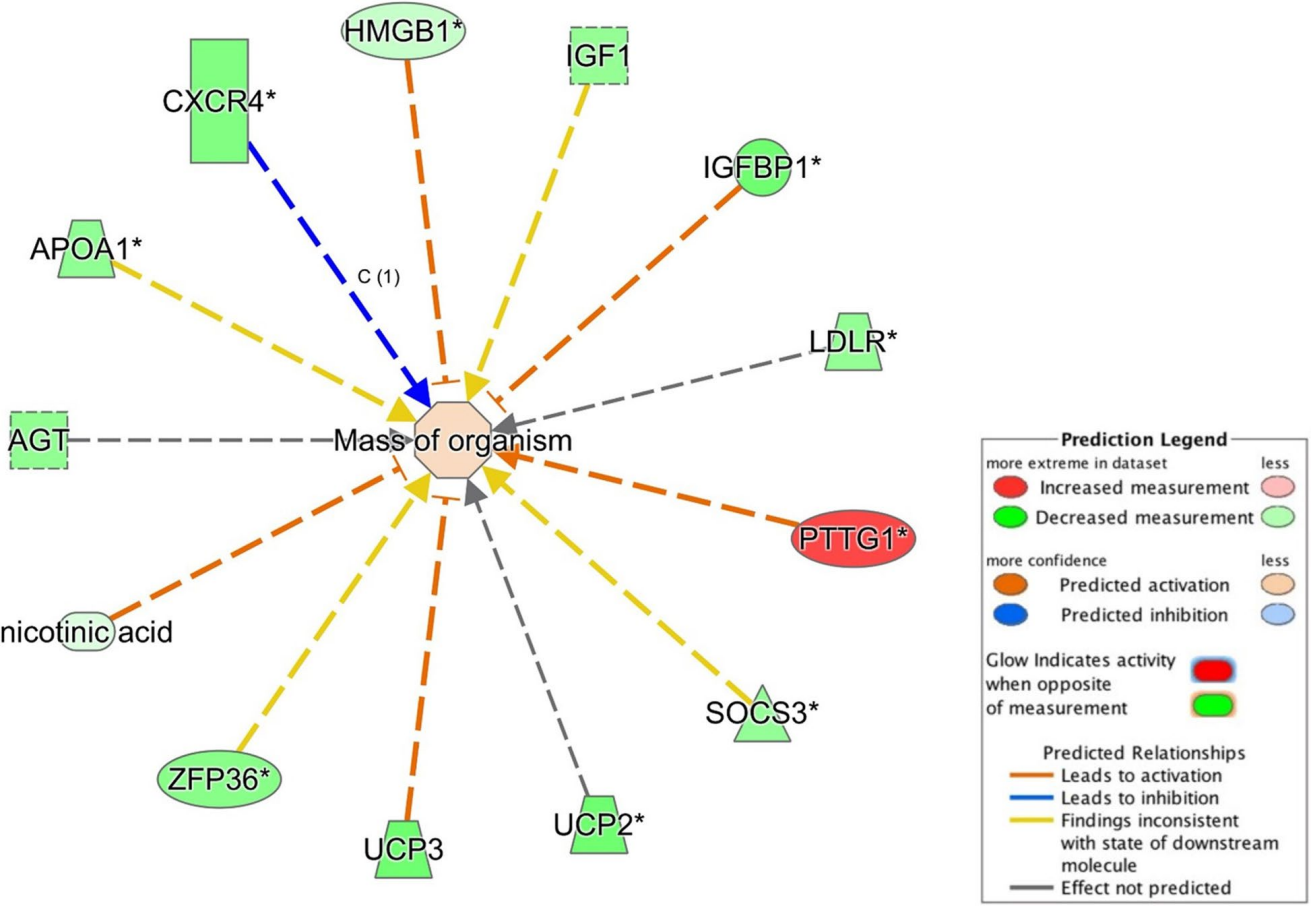
Network map showing direct relationship of one metabolite and twelve genes that were most influential to growth performance of hybrid striped bass (mass of organism). Genes and metabolites are colored by fold change: (*red*) up-regulated and (*green*) down-regulated in muscle of fish from the good-growth group. Functions based on measured gene expression and metabolite levels were predicted to be inhibited (*blue*) or activated (*orange*) in muscle of fish from the good-growth group relative to the poor-growth group. Connections between molecules and mass of organism have previously published relationships in the literature. Arrows indicate activation and perpendicular lines indicate inhibition of effect; (*orange*) and (*blue*) lines indicate agreement and (*yellow*) lines indicate disagreement with previously published literature; (*grey*) lines indicate relationships that could not be accurately concluded due to lack of current information. Image was created using Ingenuity Pathway Analysis Upstream Regulators Analysis (Qiagen IPA, Germantown, MD, USA)

Nicotinic acid (also known as niacin or vitamin B3) was considered one of the most important predictors of growth in this study (Fig. 9). Nicotinic acid is stable to thermal extrusion, however carnivorous fishes such as hybrid striped bass have a relatively high dietary requirement as they are inefficient converters of tryptophan to niacin and serotonin, the neurotransmitter antagonist to cortisol. Niacin deficiency places a higher demand on NAD(H) and NADP(H), which are required for a large number of enzymes in essentially all metabolic pathways, especially of carbohydrates, branch-chain amino acids, cholesterol, and fatty acids. The liver produces niacin from the essential amino acid tryptophan; however this synthesis is slow in carnivorous fishes. Deficiency in nicotinic acid is generally due to restricted diet and/or gastrointestinal disease. In our data, tryptophan was significantly elevated in liver of fish from the poor-growth group, and it was undetected in muscle of fish from either growth groups (Additional file 22). Although cholesterol was below threshold of detection in muscle, it was elevated in liver of fish from the poor-growth group along with related products including the bile acids cholate and taurocholate (Fig. 6). This suggests that fish from the poor-growth group were unable to convert tryptophan to niacin and/or may suffer from dietary niacin deficiency. As above with thiamin, inclusion of supplementary niacin in the prepared diet may improve growth performance of the fish.

### Pathway overview and comparison of inferential statistics and machine learning

Overall, 12 genes and one metabolite identified from muscle tissue were shown to be most influential to growth performance of hybrid striped bass (Fig. 9). This summary figure was generated based on gene expression and metabolite data and other studies of muscle growth [85–95]. These genes and metabolites are represented on the network map in Fig. 5 and together with those genes presented in Table 1 and those discussed below, provide good potential biomarkers of muscle growth in hybrid striped bass and perhaps other fishes and vertebrates as well.

**Table 1:** List of eleven alphabetically arranged hybrid striped bass muscle genes that were shared between the top 143 and 150 genes that significantly differed in expression between fish from the good- and poor- growth groups by *p*-value (*q* ≤ 0.05, FDR = 0.05) and that were chosen based on machine learning rankings, respectively. The table shows the ranking of each gene by the different approaches and the number of alternate transcript variants identified for each of the genes; more than one ranking number corresponds to the different transcript variant designations. Expression levels of all genes were up-regulated in fish from the poor-growth group based on average Fragments per Kilobase of transcript per Million mapped reads (FPKM) values

Gene Symbol	SVM Ranking	*p*-value Ranking	SVM Ranked Variants	*p*-value Ranked Variants
*agtr2*	21, 132	13, 79	2	2
*cd22*	60	33, 67	1	2
*cebpb*	117, 120	4, 5, 6, 8, 14, 101	2	6
*cxcr4*	45, 64	7, 9	2	2
*gadd45b*	70, 104	59, 107, 108	2	3
*mmp17*	4, 136	95	2	1
*msi1*	86, 102	54	2	1
*rgl1*	80, 88	12, 16, 31, 32, 99	2	5
*rgs13*	25	3	1	1
*rhou*	77	123, 124	1	2
*rundc3a*	1	56	1	1

When the muscle gene list based on statistical inference testing (FDR *q* ≤ 0.05, 143 genes) was compared to the gene list ranked by SVMAttributeEval machine learning (WEKA SMO, 150 genes), overlap of shared genes was minimal (Table 1). While there were no canonical pathways that were commonly enriched based on these 11 shared genes, there were a number of upstream regulators that showed predicted inhibition, including *tgfb1, p38*, *mapk*, and *akt* in muscle of fish from the good-growth group; one regulator, *sirt1*, was predicted to be activated in these fish and up-regulation of this gene was actually demonstrated in muscle of fish from the good-growth group as discussed above. The common functional themes of these genes echoed those aforementioned using the larger datasets: apoptosis and necrosis were predicted to influence muscle growth and were down-regulated in muscle of fish from the good-growth group and inflammation, nephritis, and insulin sensitivity were up-regulated in fish from the poor-growth group. This indicates that although individual genes detected by statistical inference-based and machine learning-based methods may differ, the overall pathways represented may still be similar. The approach used here, where both methods provided orthogonal evaluation, yielded more insight than using just a single method alone.

## Conclusions

The biological findings between expressed white muscle genes ranked using inferential statistics and machine learning identified important candidate biomarkers and pathways that influence growth performance in hybrid striped bass and perhaps other fishes and vertebrates as well. The machine learning ranked genes, when included with hundreds of measured metabolites, collectively suggested promotion of cell proliferation, cell differentiation, and bioenergetics pathways generally in muscle of fish from the good-growth group, which is in agreement with observed superior growth and muscle hyperplasia. The metabolites and genes ranked by inferential statistics provided a slightly different pathway analysis; cell proliferation and differentiation were similarly identified as activated in muscle of fish from the good-growth group, however, inflammation was also identified as an important pathway that was predominantly activated in muscle of fish from the poor-growth group. There was compelling evidence that cell death was increased in muscle of fish from the poor-growth group relative to the good-growth group, possibly related to sup-optimal growth performance or elevated cortisol levels. These pathways may reflect a precocious switch from hyperplastic to hypertrophic muscle growth as observed in fish from the poor-growth group. Overall, inferential statistics and machine learning together provided powerful orthogonal contrasts of the gene expression data, and genes provided more meaningful pathway information regarding muscle growth compared to metabolites. However, the metabolite data that more directly reflect functional aspects of the organism, did complement the gene expression findings and strengthened confirmation of energetics and inflammation pathways. Further studies assessing biomarkers known to contribute to muscle growth could provide additional criteria by which to assess changes in this and similar datasets as they become available. Several candidate biomarkers of growth are reported here, some novel and requiring further validation or testing in future research. The presented findings provide a foundation for such future studies.

## Methods

All research was performed and approved by the Institutional Animal Care and Use Committee of North Carolina State University and conducted in accordance with recommendations in the *Guide for the Care and Use of Laboratory Animals* of the National Institutes of Health [96].

### Experimental animals and tissue samples

Multiple adult fifth generation domestic striped bass males and eighth generation domestic white bass females from the *National Program for Genetic Improvement and Selective Breeding for the Hybrid Striped Bass Industry* housed at the North Carolina State University Pamlico Aquaculture Field Laboratory (NCSU, Aurora, NC, USA, 35.3054° N, 76.7885° W) were crossed *en masse* (over 100 families) to produce diverse reciprocal crossed hybrid striped bass (female white bass and male striped bass, also referred to as “sunshine” cross). These fish were reared common garden in an earthen pond (0.1 ha) and then in three outdoor flowthrough tanks (5814 L) in ambient conditions (water temperature, photoperiod) at the NCSU facility according to standard two-phase commercial-scale hybrid striped bass rearing methods [20, 21, 97]. An average cohort weight was measured at week 51.6 (conclusion of Phase 1, approximately 12 months) of culture to collect the baseline for subsequent measurement of specific growth rate (SGR). Once the majority of the cohort had reached market size (> 680 g, approximately 22 months in Phase 2) individuals from the top and bottom 10 % of the cohort by weight and total length were randomly selected as representatives of fish that grow well (*N* = 10, good-growth) and that grow poorly (*N* = 10, poor-growth), respectively. Sex of the fish was noted such that both males and females were represented in the sampled individuals, and we attempted to get close to 50% of each if possible.

Selected fish were euthanized by immersion in a solution of eugenol (AQUI-S®, Melling, Lower Hutt New Zealand) and tricaine methane sulfonate (Finquel MS-222, Argent Chemical Laboratories, Redmond, WA, USA), according to standard hatchery procedures [20, 21]. Wet weight (g) and total length (mm) data were collected for each fish. Fish were dissected and whole gonads and liver from individuals were weighed (*N* = 10 fish per group) for analysis of growth morphometrics. White muscle tissue samples were excised from the left side of each fish just ventral to the anterior margin of the first dorsal fin and posterior to the head. One muscle sample (approximately 3.5 mm^3^) from each individual (*N* = 5 fish per group) was fixed in Bouin’s fluid for three days [11] and dehydrated in 50 % alcohol for one day for muscle histology. Another muscle sub-sample (*N* = 9 fish per group) was flash frozen in liquid nitrogen and stored at -80 °C until being sent to Metabolon Inc. (Morrisville, NC, USA) for metabolomics analysis. A third muscle sub-sample (*N* = 4 fish per group) was collected and held in RNALater (Life Technologies, Carlsbad, CA, USA) overnight at 4 °C. Excess RNALater was removed the following day and samples were frozen at -80 °C until extraction of RNA and subsequent gene expression analyses. A sample of liver tissue was collected (*N* = 9 fish per group) for metabolomics analysis. Analyses were predominantly focused on muscle tissue as they can be excised in a non-lethal fashion and therefore have high potential for use in biomarker screening.

### Evaluations of growth morphometrics

Gonadosomatic index (GSI, gonad weight as a percent of total body weight in grams) and hepatosomatic index (HIS, liver weight expressed as a percent of total body weight in grams) , was calculated for each of the two growth groups [98].

Fulton’s condition factor (K) was calculated by dividing the wet weight (W) of the fish in grams by the cube length (L^3^) of the fish in centimeters, then multiplied by the factor 100 to bring K close to unity (*N* = 10 fish per group) [99].

The size of the entire hybrid striped bass cohort was measured at week 51.6 (approximately 12 months; Time_i_) by measuring a group wet weight for approximately 50 fish from each of the three rearing tanks. This value was used as Weight_i_. The specific growth rate (SGR) was calculated for fish from each growth group (*N* = 10 fish per group) based on the average group weights of fish (Weight_f_) at the end of the culturing period (Time_f_) using the following formula [100]:

$$\mathrm{SGR }=\frac{100 \times (\mathrm{ln }{{\text{Weight}}}_{{\text{f}}}-\mathrm{ln }{{\text{Weight}}}_{{\text{i}}}) }{({{\text{Time}}}_{{\text{f}}}-{{\text{Time}}}_{{\text{i}}})}$$

Statistical differences in total length, wet weight, GSI, and HSI, Fulton’s condition factor K, and SGR between the two growth groups of fish were evaluated using a One way Student’s *t*-test with an alpha level of 0.05 (SAS JMP®, 11.0.0; SAS Institute Inc., Cary, NC, USA).

### Muscle histology analysis

Fixed muscle tissues (*N* = 5 fish per group) were sent to the NCSU College of Veterinary Medicine Histology Laboratory (Raleigh, NC, USA) where they were further dehydrated through an ethanol series, embedded in paraffin, and cross-sectioned at 5 µm perpendicular to the muscle fibers. The sections were stained in haematoxylin and eosin and mounted on microscope slides for muscle morphometry analysis. Muscle fiber morphometric data were enumerated using similar methodology to Weatherley and Gill (1987) [101] and Gill et al. (1989) [102] to evaluate growth by hypertrophy (i.e., fiber area) and hyperplasia (i.e., fiber number). Representative images of muscle fibers were collected in triplicate from randomly selected areas of each tissue section (i.e., slide) for enumeration using an Olympus (Shinjuku City, Tokyo, Japan) CH light microscope (4x magnification) connected to a Celestron (Torrance, CA, USA) digital microscope camera (10x magnification). The identity of the fish corresponding to each slide was concealed from the scorer to perform blind analysis. ImageJ software (Fiji and NIH, Bethesda, MD, USA) was used to enumerate all fibers, and only those fibers that were entirely visible within the field of view were counted (i.e., those marginal fibers or those that were partially transected by the margin of view were excluded to avoid any measuring bias). The cross-sectional area of individual fibers was also calculated and, assuming the two-dimensional cross-section of muscle fibers to be circular, the diameter of each was calculated as a geometric derivative of its area.

The mean number of fibers, the mean fiber diameters (µm), were compared between the two growth groups with One-way Student’s *t*-test at an alpha level of 0.05 (SAS JMP®, 11.0.0; SAS Institute Inc., Cary, NC, USA). The presence of muscle fibers ≤ 20 µm was evaluated as an indicator of growth by hyperplasia [2] and were similarly compared between groups. In addition, a linear regression analysis was used to assess the relationship between average fiber number and average fiber diameter (µm) between the two growth groups through their corresponding average fish wet weights (g) using the linear equation: y = mx + b.

### Liver and muscle metabolomics analysis

Metabolon Inc. (Morrisville, NC, USA) performed raw data extraction, peak identification (i.e., the area-under-the-curve), and QC processing of metabolites from muscle (*N* = 9 samples per group) and liver (*N* = 9 samples per group) tissues. Briefly, the automated MicroLab STAR® system (Hamilton Company, Reno, NV, USA) was used to prepare samples. Several recovery standards were added prior to the first step in the extraction process for Quality Control (QC) purposes. To remove protein, dissociate small molecules bound to protein or trapped in the precipitated protein matrix, and to recover chemically diverse metabolites, proteins were precipitated with methanol under vigorous shaking for two minutes (Geno Grinder 2000, Glen Mills, Clifton, NJ, USA) followed by centrifugation. The resulting extract was divided into four fractions: two for analysis by two separate reverse phase (RP)/Ultrahigh Performance Liquid Chromatography-Tandem Mass Spectroscopy (UPLC-MS/MS) methods with positive ion mode electrospray ionization (ESI), one for analysis by RP/UPLC-MS/MS with negative ion mode ESI, and one for analysis by hydrophilic interaction liquid chromatography (HILIC)/UPLC-MS/MS with negative ion mode ESI. Samples were placed briefly on a TurboVap® (Zymark Corporation, Hopkinton, MA, USA) to remove the organic solvent and extracted muscle and liver tissues were stored overnight in nitrogen prior to analysis.

Identified compounds were compared to the Metabolon library of recorded purified standards or recurrent unknown entities. All identified metabolites met the retention time/index, mass to charge ratio, and chromatographic and mass spectrometry data standards of the library. Normalized values of raw area counts were recorded for each metabolite identified. Any missing values within the dataset that fell below the level of detection were scaled to the natural logarithm, such that the median was equal to one, which reigned the effects of any potential outliers. Missing values were imputed with the minimum value for a given metabolite across all muscle and liver tissue samples.

Three types of analyses were performed to identify differences in concentration of metabolites between fish from the good and poor growth groups: (1) significance tests by statistical inference (Welch’s Two Sample t-test), (2) descriptive statistics (Principal Component Analysis, PCA), and (3) random forest machine learning classification (Mean Decrease Accuracy, MDA). Standard statistical analyses were performed in ArrayStudio (Qiagen, Germantown, MD, USA) on log transformed data. For those analyses not standard in ArrayStudio, the programs R 3.5.0 or SAS JMP 11.0.0 were used. Welch’s Two-sample t-test was used on liver and muscle tissue samples to determine significant differences in metabolite concentrations between the two growth groups. A PCA was conducted to identify if the fish samples from the poor-growth and good-growth groups could be separated based solely on the metabolic signatures. To determine which of the identified metabolites made the largest contribution to the classification, MDA was computed as a sensitivity measure; the MDA was determined by a random forest machine learning analysis that provided an ordered list of the top thirty metabolites ranked by importance in classification.

### Muscle gene expression sequencing

The NCSU Genomic Sciences Laboratory (Raleigh, NC, USA) performed RNA extraction and library preparation for RNA-Seq via the NexSeq 500 platform (Illumina, San Diego, CA, USA). Total RNA was extracted using an RNeasy Fibrous Tissue mini total RNA isolation kit and manufacturer’s protocol (Qiagen). The libraries were sequenced using 75 bp single read chemistry and samples were pooled into one-half of a lane for approximately 23 to 30 million reads per sample. Prior to library construction, RNA integrity, purity, and concentration were assessed using a 2100 Bioanalyzer with an RNA 6000 Nano Chip (Agilent Technologies, Santa Clara, CA, USA). Purification of messenger RNA (mRNA) was performed using the oligo-dT beads provided in the NEBNext Poly(A) mRNA Magnetic Isolation Module (New England Biolabs, Ipswich, MA, USA). Complementary DNA (cDNA) libraries for Illumina sequencing were constructed using the NEBNext Ultra Directional RNA Library Prep Kit and NEBNext Mulitplex Oligos for Illumina using the manufacturer-specified protocol. Briefly, the mRNA was chemically fragmented and primed with random oligos for first strand cDNA synthesis. Second strand cDNA synthesis was then carried out with dUTPs to preserve strand orientation information. The double-stranded cDNA was then purified, end repaired and A-tailed for adaptor ligation. Following ligation, the samples were selected for final library size (adapters included) of 400-550 bp using sequential AMPure XP bead isolation (Beckman Coulter, Brea, CA, USA). Library enrichment was performed and specific indexes for each sample were added during the protocol-specified PCR amplification. The amplified library fragments were purified and checked for quality and final concentration using an Agilent 2100 Bioanalyzer with a High Sensitivity DNA chip. The final quantified libraries were pooled in equimolar amounts for sequencing and flow cell cluster generation on the llumina NextSeq 500 DNA sequencer utilizing 75 bp single read sequencing with NextSeq Reagent Kit v2.

### Inferential statistics evaluation of muscle gene expression

RNA-Seq data analysis was performed by Data2Bio (Ames, IA, USA). Prior to alignment, the nucleotides of each single end raw read Illumina NexSeq read were scanned for low quality. Bases with PHRED quality value < 20 out of 40 [103, 104] were removed and only those with error rates of ≤ 1 % were included. Each read was examined in two phases. In the first phase, reads were scanned starting at each end and nucleotides with quality values lower than the PHRED threshold were removed. The remaining nucleotides were then scanned using overlapping windows of 10 bp and sequences with an average PHRED quality value less than the specified threshold beyond the last window were truncated. Trimming parameters were in reference to the software LUCY2 [105, 106] and trimmed reads were aligned to the annotated reference striped bass genome (NCBI GenBank: GCA_001663605.1) using GSNAP [107]. Confidently mapped, single end reads were filtered if they were mapped uniquely (≤ 2 mismatches every 36 bp and less than 5 bp for every 75 bp as tails) and used for subsequent analyses. The coordinates of uniquely aligned reads to the reference genome were used for positional reference and read count tallies were computed for each annotated gene. Singleton reads were assigned a count of one when their aligned coordinates overlapped with an annotated gene. Normalization was conducted by Bioconductor DESeq2 1.28.1 [108], which corrects for biases introduced by differences in the total numbers of uniquely mapped reads in each sample and that also partially corrects for SNP variation between genotypes (i.e., biological variation among fish). Normalized read counts were used to calculate fold-changes and statistical significance. The R package DESeq2 was used to test the null hypothesis that expression of a given gene is not different between the two growth performance groups of poor and good [108]. A model using the negative binomial distribution of read counts was used to test this null hypothesis by statistical inference. The read counts per gene obtained from all the samples were used for the differential expression analysis. Before conducting the test for differentially expressed genes, a PCA was conducted with the DESeq2 package using the top 500 genes with highest variance among all samples to assay the quality of these samples. The *p*-values of all DESeq2 statistical tests were converted to adjusted *p*-values (*q*-values) based on false discovery rate (FDR) [109] and an FDR of 5 % (*q*-value) was used to account for multiple testing; 143 genes met this criterion of *q* ≤ 0.05 and were used in pathway analysis (see “Combinatorial Muscle Pathway Analysis” section below).

### Machine learning evaluation of muscle gene expression

Machine learning models were used to evaluate muscle gene expression in hybrid striped bass related to the good- and poor-growth groups. All analyses were performed using WEKA version 3.8 (University of Waikato, Hillcrest, New Zealand) [110] and generally follow our previously published approach [111–117]. The Sequential Minimal Optimization Support Vector Machines (SMO) was used to predict hybrid striped bass growth performance (classified as either good- or poor-growth) based on expression of muscle gene transcripts (Fragments per Kilobase of Transcript per Million Mapped Reads; FPKM values). Briefly, data from a subset of the fish were used to train each machine learning model and then the remaining data were used to cross-validate the learned patterns. During this cross-validation, the model predicts the growth performance of each individual fish (good- or poor-growth group assignment) based on gene expression patterns that it has learned during the training step. Two cross-validation strategies were used to evaluate the learning of each model: (1) a percentage split whereby 66 % of the data were randomly selected and used to train the models and the remaining 34 % of the data were input as a cross-validation and (2) a 8-fold stratified hold out with *n* = 8 folds where one fold was used for cross-validation and n – 1 folds of the randomly reordered data set were used for training. Both classes (good- and poor-growth groups) were equally represented in the model training and cross-validation data to avoid bias. The percentages of correct class assignments during cross-validation (i.e., predictive accuracy) were used to evaluate model robustness [118] along with the Kappa statistic and area under the receiver operating characteristic curve (AUROC) [111].

The 72893 expressed muscle gene transcripts deemed informative through DESeq2 were included in initial SMO machine learning modeling to predict hybrid striped bass growth group based on FPKM values, which were then subsequently ranked by importance of information during the model-training step using SVMAttributeEval [111, 119]. The SVMAttributeEval ranking procedure is a sensitivity analysis that allows for reduction of data dimensionality, a process to omit genes that are not assigned an information rank and to identify only those genes that are most important for differentiating between fish of the good- and poor-growth groups.

Further reduction of data dimensionality occurred in two orthogonal steps: an evaluation of model overfitting, defined as too many input gene attributes, and an evaluation of model underfitting, defined as too few. To evaluate model overfitting, the ranked gene list was used to evaluate the performance of a series of different SMO models using subsets of the top highly ranked genes as inputs (i.e., the top 10, 25, 50, 75, 100, 150, 250, 300, 400, 500, 600, 700, 800, 900, 1000, 2000, 3000, 4000, 5000, 6000, 7000, 8000, 9000, 10000, 20000, 30000, 40000, 50000, 60000, and 70000 genes). The percentage of correctly classified instances for each of these models were plotted against the number of top highly ranked input gene values and each plot was fitted with a polynomial trendline of an order two or three (i.e., the maximum number of input gene values that still provided optimal model performance was plotted to visualize and determine overfitting of training models). Analyzing the best fit of a model allowed for the identification of the maximum number of input genes and the most important ones in relation to the classification question.

To evaluate model underfitting (i.e., to identify the minimum number of input gene values that still provided optimal model performance), the top 10000 ranked genes were used as baseline for SMO model performance and then the top 10, 25, 50, 75, 100, 150, 250, 500, 1000, 2000, 3000, 4000, 5000, 6000, 7000, 8000, 9500, 9600, 9700, 9800, 9900, 9990, 9995, 9996, 9997, 9998, and 9999 gene values were subsequently eliminated from the gene input lists for another series of SMO models performed otherwise as described above. These reduced gene lists confirmed the importance of including at least the top 150 ranked genes as inputs to optimally predict fish growth and were chosen for pathway analysis (see “Combinatorial Muscle Pathway Analysis” section below).

The performance of all machine learning models was interpreted in comparison to appropriate negative controls for learning. One negative control was based on the Law of Probability, an assumption that randomly classifying fish into one of two groups (i.e., good- or poor-growth) would be achieved with a success rate of 50 % based on random chance alone. The second negative control was conducted by randomizing the data labels according to each independent variable used for the SMO machine learning models and entering them into new models as described above. A total of eight such iterations of training and cross-validation using different, randomly ordered datasets was conducted for each negative control and the average performance was recorded [111, 112].

### Combinatorial muscle pathway analysis

To ascertain biological processes that may underlie fish growth performance differences, a combinatorial Qiagen Ingenuity Pathway Analysis (IPA) was conducted using both the metabolites and differentially expressed genes measured in hybrid striped bass muscle [120]. The 469 muscle metabolites identified from the global quantitative metabolomics panel analysis were mapped against the IPA KnowledgeBase using three identifiers (KEGG, HMDB, and PUBCHEM ID). Two gene lists 1) genes with a *q*-value of differential expression at ≤ 0.05 cutoff (DEseq2 statistical inference) (143 genes) and 2) genes top-ranked by SVMAttributeEval (WEKA machine learning) (150 genes) were mapped to known human ortholog gene identities for use in IPA. Two pathway *Core Analyses* were conducted, each using one of the two mapped gene lists paired with the list of 469 metabolites. Comparison results were marked for expression by log2 fold-change values and *p*-value to be used as a cutoff in the analysis. A *p*-value of 0.05 was used as a filter cutoff for metabolites; no filter was applied to the gene lists, as the one ranked by inferential statistics already met the *q*-value cutoff of 0.05 and a *p*-value cutoff is meaningless for the machine learning ranked gene list as it is a non-statistical evaluation. All molecules detected on each platform, regardless of inclusion in pathway annotation or passage of applied cutoffs (e.g., by *p*-value) were included in analyses as a reference set (i.e., 469 metabolites and 72893 expressed genes).

The Qiagen Ingenuity Knowledge Base, a data repository of interactions, annotations, and other queries regarding the relationships between genes and other molecules was used to categorize pathways and functions of these data. In IPA *Core Analyses*, the enrichment of a pathway or function is identified by the number of observed significant differentially expressed molecules assigned to that pathway or function. *Canonical Pathway Analysis* was used to detect enrichment of pathways based on observed differences in gene expression and metabolite levels within each pathway and *Molecule Activity Predictor* (MAP) was overlain to predict activity patterns of the pathways; *Upstream Regulators Analysis* was used to predict causal effects of upstream regulator proteins and their downstream genes derived from existing scientific literature and based on observed differences in gene expression and metabolite levels; and *Diseases and Functions Analysis* was used to predict causal effects including cellular processes and biological functions derived from existing literature and that are expected to change based on observed differences in expressed gene and metabolites levels. The Analytical Analysis tool *BioProfiler* was conducted independently and filtered to the phenotypes of interest, including growth of muscle and response of liver. *BioProfiler* was used to make novel discoveries by providing the ability to filter fine-grained relationships between molecules (genes and metabolites) and diseases or functions.

The Fisher’s Exact Test at an alpha level of 0.05 was used to calculate statistical significance of pathway effects. The test evaluates the number of molecules that are in the reference set and those that 1) match between a pathway annotation and those that passed any applied cutoffs (metabolites only, *p* ≤ 0.05); 2) are associated with the pathway annotation, but did not pass applied cutoffs, and 3) that passed applied cutoffs, but did not match the pathway annotation molecules. The reference set included all molecules that were possible to assay in the experiment (i.e., that were detectable on each platform used), but were not included in the pathway annotation or did not pass applied cutoffs (i.e., of the 469 metabolites and 72893 expressed genes). In the right-tailed Fisher's Exact Test, only over-represented pathway annotations (i.e., those that were represented by more molecules than expected by chance) are considered significant. Under-represented annotations (“left-tailed” *p*-values), which have significantly fewer molecules than expected by chance, are not considered enriched or over-represented and were not reported.

### Supplementary Information

**Supplementary Material 1.**

**Supplementary Material 2.**

**Supplementary Material 3.**

**Supplementary Material 4.**

**Supplementary Material 5.**

**Supplementary Material 6.**

**Supplementary Material 7.**

**Supplementary Material 8.**

**Supplementary Material 9.**

**Supplementary Material 10.**

**Supplementary Material 11.**

**Supplementary Material 12.**

**Supplementary Material 13.**

**Supplementary Material 14.**

**Supplementary Material 15.**

**Supplementary Material 16.**

**Supplementary Material 17.**

**Supplementary Material 18.**

**Supplementary Material 19.**

**Supplementary Material 20.**

**Supplementary Material 21.**

**Supplementary Material 22.**

**Supplementary Material 23.**

**Supplementary Material 24.**

**Supplementary Material 25.**

**Supplementary Material 26.**

**Supplementary Material 27.**

**Supplementary Material 28.**

**Supplementary Material 29.**

**Supplementary Material 30.**

**Supplementary Material 31.**

**Supplementary Material 32.**

**Supplementary Material 33.**

## Data Availability

All metabolite data are published as Additional files 3 and 4. Gene expression data are published as Additional file 8 and as sequence files under NCBI BioProject Accession: PRJNA950439.
