# Supplementary material for: Combinatorial metabolomic and transcriptomic analysis of muscle growth in hybrid striped bass (female white bass Morone chrysops x male striped bass M. saxatilis)
Source: BMC Genomics. 2024 Jun 10;25:580. doi: 10.1186/s12864-024-10325-y (PMC11165755; doi:10.1186/s12864-024-10325-y)
Supplement: Supplementary file 1 — Supplementary Material 1. [file 12864_2024_10325_MOESM1_ESM.docx]

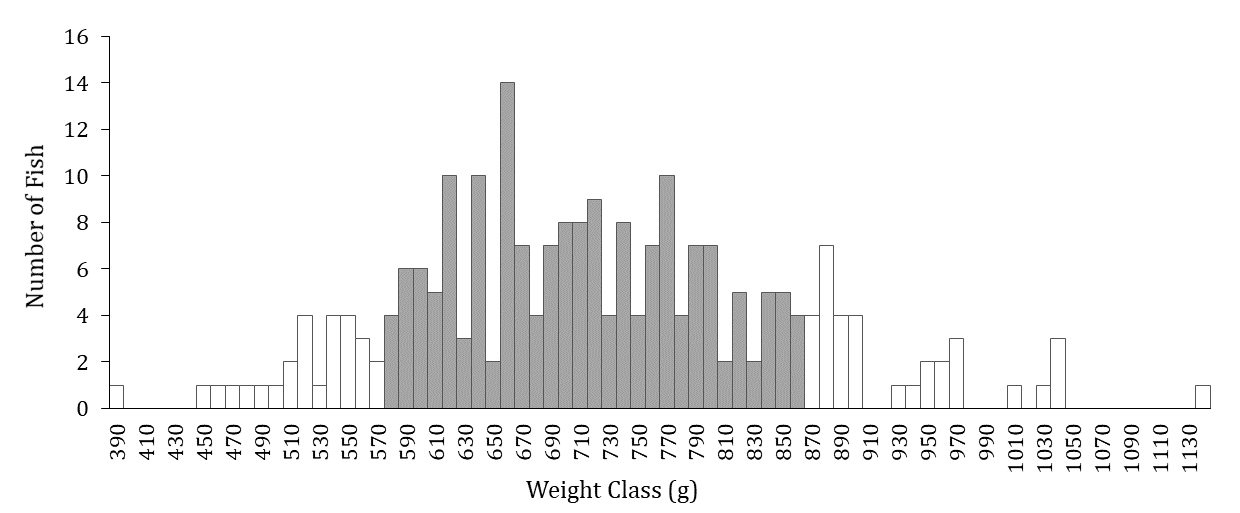


**Additional File 1 (Supplemental Figure 1).** Bar charts showing the weight class (g) frequency in numbers of all hybrid striped bass from the rearing cohort. The white bars represent the top and bottom 10 % of fish in terms of body size and N = 10 fish each were selected as representatives of fish that grow good (good-growth) or poor (poor-growth), respectively, from within these ranges. These cutoffs were chosen as they represent a body size that is typically larger and smaller than that desired at market and therefore represent the extremes of the distribution.
