# Supplementary material for: Combinatorial metabolomic and transcriptomic analysis of muscle growth in hybrid striped bass (female white bass Morone chrysops x male striped bass M. saxatilis)
Source: BMC Genomics. 2024 Jun 10;25:580. doi: 10.1186/s12864-024-10325-y (PMC11165755; doi:10.1186/s12864-024-10325-y)
Supplement: Supplementary file 2 — Supplementary Material 2. [file 12864_2024_10325_MOESM2_ESM.docx]

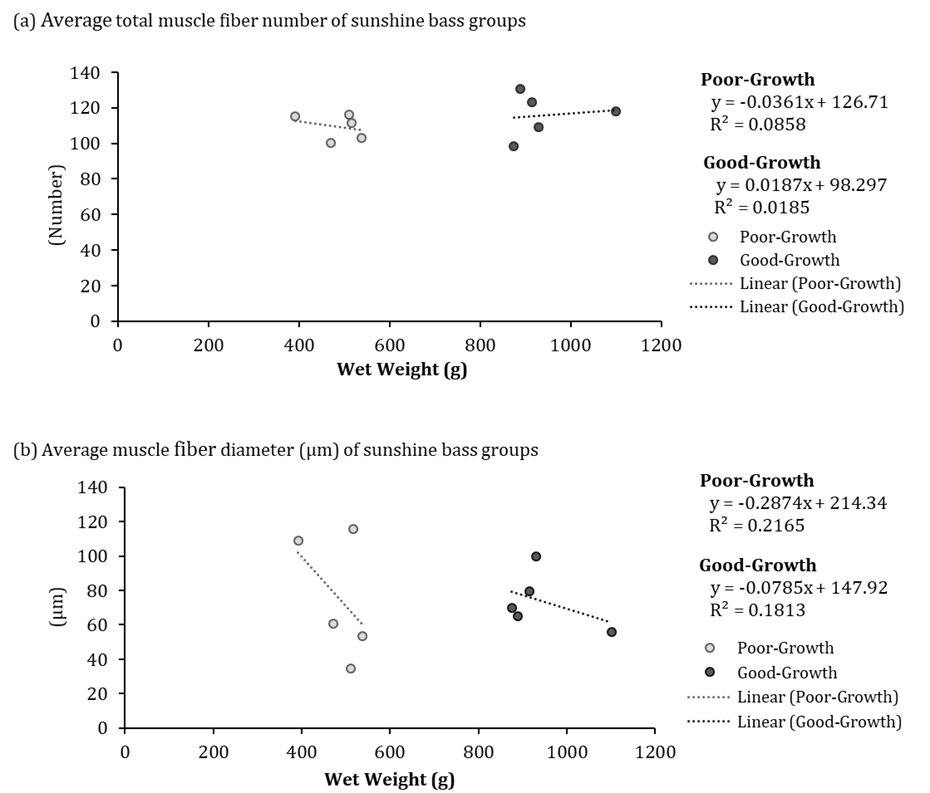


**Additional File 2 (Supplemental Figure 2).** Linear regression graphs of the average total muscle fiber number and hybrid striped bass wet weight (g) of the poor- and good- growth groups. For each growth group, the linear regression equation is given as y = mx + b, where the slopes (m) of the lines represent the linear correlation between the variables in each growth groups; correlation coefficient (R^2^) is provided. The linear correlation between average total muscle fiber number and fish wet weight of the good- and poor-growth groups showed a weak correlation (R^2^=0.0858 and 0.0185, respectively) and the linear correlation between the average muscle fiber diameter and fish wet weight of the good- and poor-growth groups showed a weak correlation (R^2^=0.1813 and 0.2165, respectively).
