# Supplementary material for: Combinatorial metabolomic and transcriptomic analysis of muscle growth in hybrid striped bass (female white bass Morone chrysops x male striped bass M. saxatilis)
Source: BMC Genomics. 2024 Jun 10;25:580. doi: 10.1186/s12864-024-10325-y (PMC11165755; doi:10.1186/s12864-024-10325-y)
Supplement: Supplementary file 5 — Supplementary Material 5. [file 12864_2024_10325_MOESM5_ESM.docx]

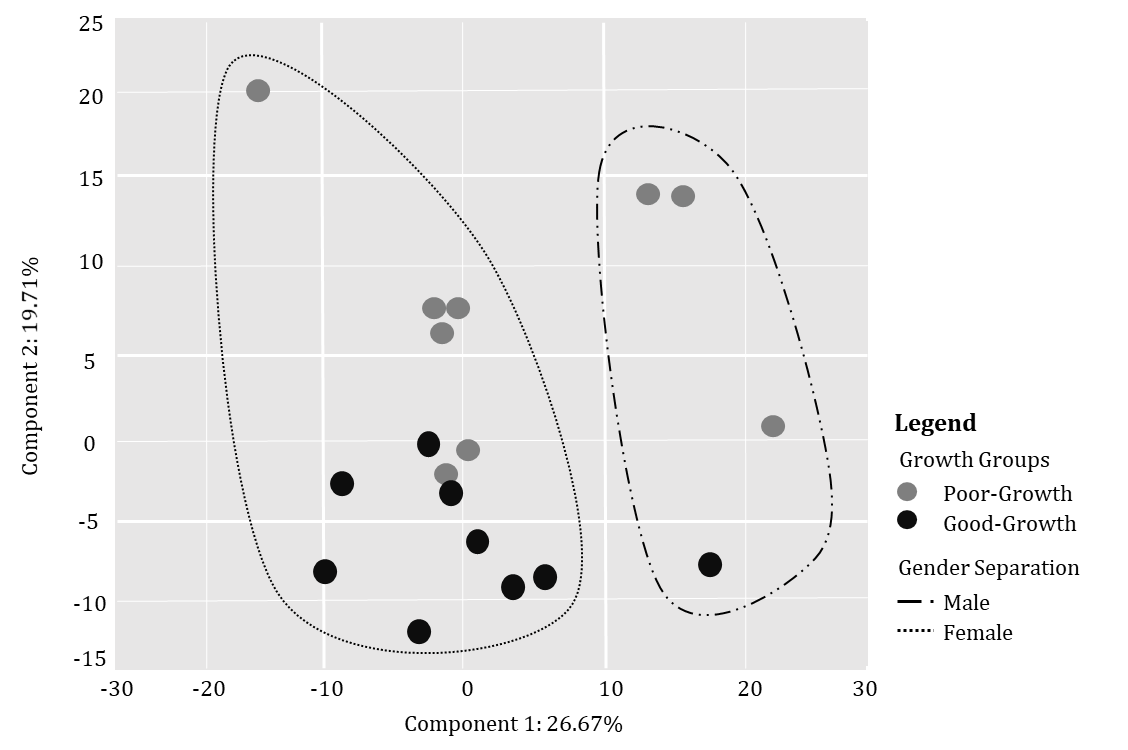


**Additional File 5 (Supplemental Figure 3).** Principle Component Analysis (PCA) based on 464 metabolite concentrations in hybrid striped bass liver tissue of fish from the good- and poor-growth groups. Component 2 appeared to differentiate the two growth groups of fish.
