# Supplementary material for: Combinatorial metabolomic and transcriptomic analysis of muscle growth in hybrid striped bass (female white bass Morone chrysops x male striped bass M. saxatilis)
Source: BMC Genomics. 2024 Jun 10;25:580. doi: 10.1186/s12864-024-10325-y (PMC11165755; doi:10.1186/s12864-024-10325-y)
Supplement: Supplementary file 6 — Supplementary Material 6. [file 12864_2024_10325_MOESM6_ESM.docx]

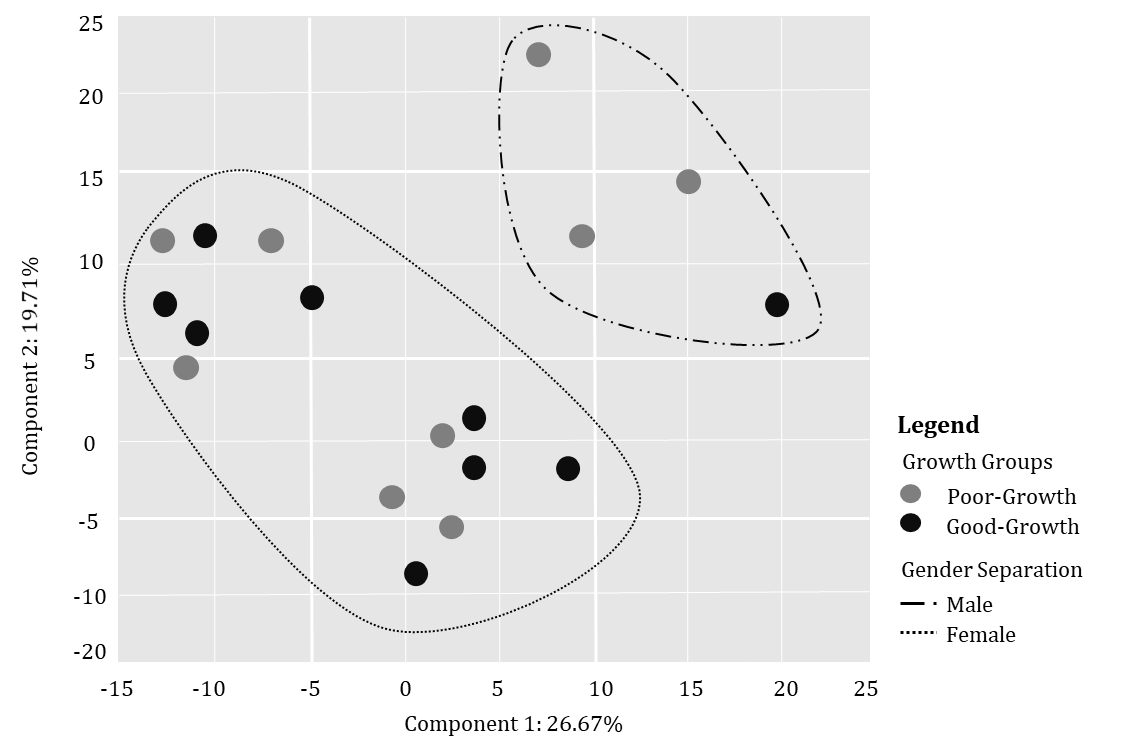


**Additional File 6 (Supplemental Figure 4).** Principle Component Analysis (PCA) based on 469 metabolite concentrations in hybrid striped bass muscle tissue of fish from the good- and poor-growth groups. Neither component 1 nor 2 appeared to clearly differentiate the two growth groups of fish.
