# Supplementary material for: Combinatorial metabolomic and transcriptomic analysis of muscle growth in hybrid striped bass (female white bass Morone chrysops x male striped bass M. saxatilis)
Source: BMC Genomics. 2024 Jun 10;25:580. doi: 10.1186/s12864-024-10325-y (PMC11165755; doi:10.1186/s12864-024-10325-y)
Supplement: Supplementary file 7 — Supplementary Material 7. [file 12864_2024_10325_MOESM7_ESM.docx]

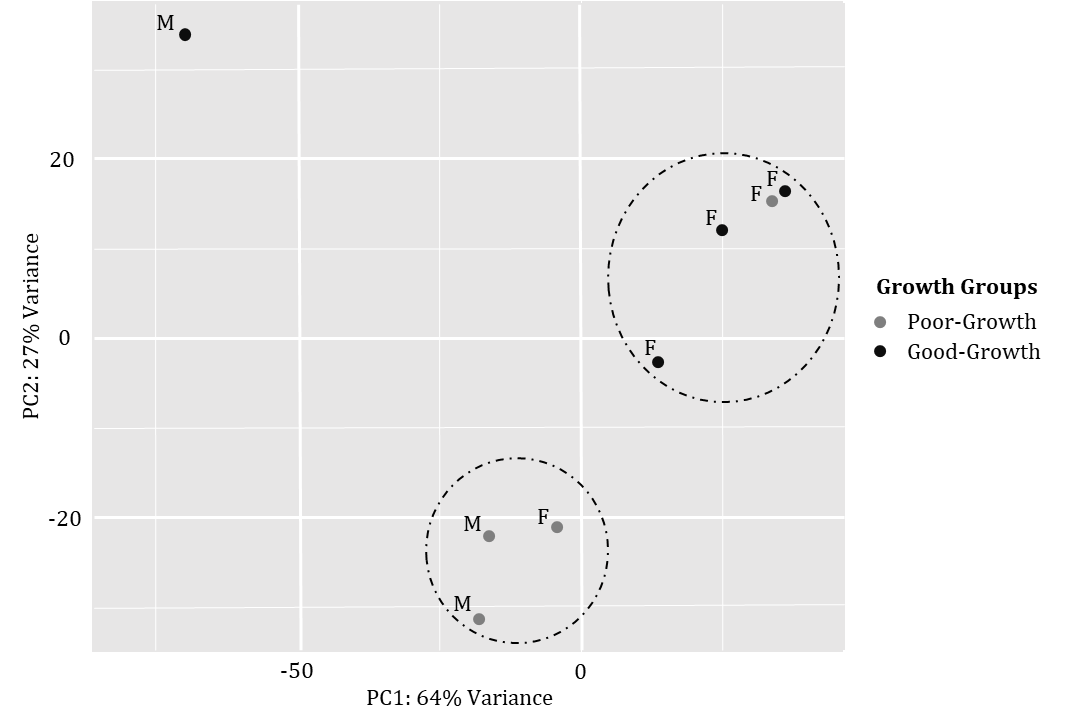


**Additional File 7 (Supplemental Figure 5).** Principal Component Analysis (PCA) using top 500 highest variance expressed genes in white muscle samples of hybrid striped bass. The data were clustered together based on growth performance of the hybrid striped bass, where the light grey color points show the poor-growth group, and the dark grey points show the good-growth group; M and F indicate male and female gender, respectively. PC1 and PC2 are the parameters that comprise these 500 genes and together they explain 91% of the variation in fish growth performance.
