# Supplementary material for: Combinatorial metabolomic and transcriptomic analysis of muscle growth in hybrid striped bass (female white bass Morone chrysops x male striped bass M. saxatilis)
Source: BMC Genomics. 2024 Jun 10;25:580. doi: 10.1186/s12864-024-10325-y (PMC11165755; doi:10.1186/s12864-024-10325-y)
Supplement: Supplementary file 9 — Supplementary Material 9. [file 12864_2024_10325_MOESM9_ESM.docx]

**Additional File 9 (Supplemental Table 4).** List of the top 143 most important expressed genes in white skeletal muscle of hybrid striped bass that significantly differed in expression between fish from the poor- and good- growth groups (genes are ranked based on significance q ≤ 0.05, FDR = 0.05). Differences in elevated expression level between the two growth groups (i.e., poor- and good-growth) is expressed as the average FPKM value (Fragments per Kilobase of Transcript per Million Mapped Reads). Up-regulated genes between the two growth groups are indicated in bold-faced text. The variant designation indicates that several genes were identified more than once, such that different *de novo* transcript assembles corresponding to the same gene were identified (e.g., spliced variants or allelic variants originating from the striped bass and white bass genomes as expressed in the hybrid striped bass).

Average FPKM

Poor- Good-

Rank and Gene Name Gene Symbol Growth Growth

1 mRNA decay activator protein ZFP36 (Variant 1) *zfp36* **14.100** 5.750

2 mRNA decay activator protein ZFP36 (Variant 2) *zfp36* **23.486** 10.207

3 Regulator of G Protein Signaling 13 *rgs13* **3.866** 0.579

4 CCAAT/Enhancer Binding Protein Beta (Variant 1) *cebpb* **3.408** 0.884

5 CCAAT/Enhancer Binding Protein Beta (Variant 2) *cebpb* **3.408** 0.884

6 CCAAT/Enhancer Binding Protein Beta (Variant 3) *cebpb* **2.940** 0.813

7 C-X-C Motif Chemokine Receptor 4 (Variant 1) *cxcr4* **4.881** 1.609

8 CCAAT/Enhancer Binding Protein Beta (Variant 4) *cebpb* **5.282** 1.396

9 C-X-C Motif Chemokine Receptor 4 (Variant 2) *cxcr4* **2.834** 0.949

10 Sphingosine-1-Phosphate Receptor 1 (Variant 1) *s1pr1* **1.912** 0.563

11 mRNA decay activator protein ZFP36 (Variant 3) *zfp36* **17.528** 8.409

12 Ral Guanine Nucleotide Dissociation Stimulator Like 1 (Variant 1) *rgl1* **3.043** 1.313

13 Angiotensin II Receptor Type 2 (Variant 1) *agtr2* **1.260** 0.614

14 CCAAT/Enhancer Binding Protein Beta (Variant 5) *cebpb* **5.877** 1.521

15 Suppressor Of Cytokine Signaling 3 (Variant 1) *socs3* **2.244** 0.932

16 Ral Guanine Nucleotide Dissociation Stimulator Like 1 (Variant 2) *rgl1* **1.674** 0.723

17 Insulin Like Growth Factor Binding Protein 1 (Variant 1) *igfbp1* **2.012** 0.385

18 Mitochondrial uncoupling protein 2 (Variant 1) *ucp2* **190.569** 31.935

19 Uncoupling Protein 3 *ucp3* **70.482** 11.856

20 Myogenin *myog* 0.858 **4.258**

21 Mitochondrial uncoupling protein 2 (Variant 2) *ucp2* **37.850** 6.504

22 Insulin Like Growth Factor Binding Protein 1 (Variant 2) *igfbp1* **6.012** 1.378

23 Sphingosine-1-Phosphate Receptor 1 (Variant 2) *s1pr1* **1.941** 0.556

24 Mitochondrial uncoupling protein 2 (Variant 3) *ucp2* **45.623** 8.143

25 Mitochondrial uncoupling protein 2 (Variant 4) *ucp2* **45.623** 8.143

26 Rho Family GTPase 3 (Variant 1) *rnd3* **3.942** 1.469

27 Rho Family GTPase 3 (Variant 2) *rnd3* **3.942** 1.469

28 Insulin Like Growth Factor Binding Protein 1 (Variant 3) *igfbp1* **4.628** 1.117

29 Regulator Of G Protein Signaling 21 *rgs21* **1.628** 0.446

30 Nudix Hydrolase 16 *nudt16* **0.963** 0.248

31 Ral Guanine Nucleotide Dissociation Stimulator Like 1 (Variant 3) *rgl1* **6.481** 3.842

32 Ral Guanine Nucleotide Dissociation Stimulator Like 1 (Variant 4) *rgl1* **3.196** 1.365

33 CD22 Molecule (Variant 1) *cd22* **0.321** 0.129

34 Rho Family GTPase 3 (Variant 3) *rnd3* **4.235** 1.587

35 High Mobility Group Box 1 (Variant 1) *hmgb1* **33.082** 23.954

36 High Mobility Group Box 1 (Variant 2) *hmgb1* **32.859** 23.792

37 High Mobility Group Box 1 (Variant 3) *hmgb1* **32.859** 23.792

38 Apolipoprotein A1 (Variant 1) *apoa1* **18.656** 7.704

39 Apolipoprotein A1 (Variant 2) *apoa1* **13.971** 5.786

40 Glutamate-Ammonia Ligase (Variant 1) *glul* **10.029** 3.659

41 Glutamate-Ammonia Ligase (Variant 2) *glul* **7.402** 2.580

42 Serpin Family A Member 1 (Variant 1) *serpina1* **1.793** 0.667

43 C-X-C Motif Chemokine Ligand 8 *cxcl8* **1.533** 0.644

44 Angiotensinogen *agt* **1.394** 0.519

45 Thrombospondin 1 (Variant 1) *thbs1* **32.892** 14.735

46 Thrombospondin 1 (Variant 2) *thbs1* **12.900** 5.619

47 Glutamate-Ammonia Ligase (Variant 3) *glul* **11.832** 4.138

48 Glutamate-Ammonia Ligase (Variant 4) *glul* **11.832** 4.138

49 Serpin Family A Member 1 (Variant 2) *serpina1* **2.457** 0.926

50 Serpin Family A Member 1 (Variant 3) *serpina1* **2.251** 0.845

51 Arrestin Domain Containing 2 (Variant 1) *arrdc2* **84.926** 18.076

52 Arrestin Domain Containing 2 (Variant 2) *arrdc2* **84.926** 18.076

53 Glutamate-Ammonia Ligase (Variant 5) *glul* **10.028** 3.700

54 Musashi RNA Binding Protein 1 *msi1* **4.717** 2.792

55 Diablo IAP-Binding Mitochondrial Protein (Variant 1) *diablo*  **1.653** 0.172

56 RUN Domain Containing 3A *rundc3a* **5.779** 3.751

57 Arrestin Domain Containing 2 (Variant 3) *arrdc2* **121.441** 25.657

58 Arrestin Domain Containing 2 (Variant 4) *arrdc2* **121.441** 25.657

59 Growth Arrest And DNA Damage Inducible Beta (Variant 1) *gadd45b* **1.084** 0.449

60 Aldolase, Fructose-Bisphosphate B (Variant 1) *aldob* **5.415** 3.235

61 Delta Like Canonical Notch Ligand 4 (Variant 1) *dll4* **16.781** 3.509

62 Glutamate-Ammonia Ligase (Variant 6) *glul* **10.638** 3.510

63 PDZ And LIM Domain 5 *pdlim5* **0.669** 0.181

64 Aldolase, Fructose-Bisphosphate B (Variant 2) *aldob* **4.081** 2.373

65 Aldolase, Fructose-Bisphosphate B (Variant 3) *aldob* **4.062** 2.350

66 Aldolase, Fructose-Bisphosphate B (Variant 4) *aldob* **3.265** 1.898

67 CD22 Molecule (Variant 2) *cd22* **1.138** 0.511

68 Arrestin Domain Containing 2 (Variant 5) *arrdc2* **100.600** 21.330

69 Arrestin Domain Containing 2 (Variant 6) *arrdc2* **100.600** 21.330

70 Arrestin Domain Containing 2 (Variant 7) *arrdc2* **100.232** 21.274

71 Glutamate-Ammonia Ligase (Variant 7) *glul* **7.229** 2.470

72 Aldolase, Fructose-Bisphosphate B (Variant 5) *aldob* **3.735** 2.169

73 Aldolase, Fructose-Bisphosphate B (Variant 6) *aldob* **3.537** 2.056

74 Aldolase, Fructose-Bisphosphate B (Variant 7) *aldob* **3.471** 2.018

75 Pituitary Tumor-Transforming 1 (Variant 1) *pttg1* 0.424 **2.876**

76 Pituitary Tumor-Transforming 1 (Variant 2) *pttg1* 0.415 **2.820**

77 Pituitary Tumor-Transforming 1 (Variant 3) *pttg1* 0.313 **2.125**

78 Pituitary Tumor-Transforming 1 (Variant 4) *pttg1* 0.309 **2.094**

79 Angiotensin II Receptor Type 2 (Variant 2) *agtr2* **1.294** 0.690

80 Serpin Family A Member 1 (Variant 4) *serpina1* **1.319** 0.490

81 Serpin Family A Member 1 (Variant 5) *serpina1* **1.301** 0.484

82 Serpin Family A Member 1 (Variant 6) *serpina1* **1.238** 0.460

83 Serpin Family A Member 1 (Variant 7) *serpina1* **1.235** 0.459

84 Arrestin Domain Containing 2 (Variant 8) *arrdc2* **97.687** 20.735

85 PRELI Domain Containing 3B *prelid3b* **8.606** 4.103

86 Fibrinogen Beta Chain (Variant 1) *fgb* **4.256** 1.451

87 Aldolase, Fructose-Bisphosphate B (Variant 8) *aldob* **4.493** 2.645

88 Regulator Of G Protein Signaling 5 *rgs5* **0.254** 0.073

89 Phosphoenolpyruvate Carboxykinase 1 *pck1* **0.470** 0.094

90 Arrestin Domain Containing 2 (Variant 9) *arrdc2* **50.844** 11.077

91 Arrestin Domain Containing 2 (Variant 10) *arrdc2* **50.844** 11.077

92 TSC22 Domain Family Member 3 (Variant 1) *tsc22d3* **32.676** 14.439

93 Apolipoprotein A1 (Variant 3) *apoa1* **7.028** 3.097

94 Early Growth Response 1 *egr1* **1.751** 0.780

95 Matrix Metallopeptidase 17 *mmp17* **2.056** 0.885

96 Delta Like Canonical Notch Ligand 4 (Variant 2) *dll4* **89.949** 15.985

97 Growth Arrest And DNA Damage Inducible Alpha (Variant 1) *gadd45a* **1.547** 0.608

98 Growth Arrest And DNA Damage Inducible Alpha (Variant 2) *gadd45a* **1.541** 0.606

99 Ral Guanine Nucleotide Dissociation Stimulator Like 1 (Variant 5) *rgl1* **7.190** 4.522

100 TSC22 Domain Family Member 3 (Variant 2) *tsc22d3* **38.958** 17.163

101 CCAAT/Enhancer Binding Protein Beta (Variant 6) *cebpb* **2.718** 1.003

102 Fatty Acid Binding Protein 1 *fabp1* **4.577** 0.854

103 Pituitary Tumor-Transforming 1 (Variant 5) *pttg1* 0.244 **1.393**

104 Arrestin Domain Containing 2 (Variant 11) *arrdc2* **40.232** 9.866

105 Growth Arrest And DNA Damage Inducible Gamma (Variant 1) *gadd45g* **66.373** 27.044

106 Arrestin Domain Containing 2 (Variant 12) *arrdc2* **31.104** 7.642

107 Growth Arrest And DNA Damage Inducible Beta (Variant 2) *gadd45b* **2.413** 0.975

108 Growth Arrest And DNA Damage Inducible Beta (Variant 3) *gadd45b* **2.302** 0.930

109 Solute Carrier Family 3 Member 2 (Variant 1) *slc3a2* **60.590** 18.032

110 Solute Carrier Family 3 Member 2 (Variant 2) *slc3a2* **48.054** 14.302

111 Growth Arrest And DNA Damage Inducible Gamma (Variant 2) *gadd45g* **53.649** 21.904

112 Growth Arrest And DNA Damage Inducible Gamma (Variant 3) *gadd45g*  **53.649** 21.904

113 Low Density Lipoprotein Receptor (Variant 1) *ldlr*  **2.192** 0.933

114 Flavin Containing Monooxygenase 5 *fmo5* **0.485** 0.082

115 Arginine Vasopressin Induced 1 *avpi1* **5.048** 2.330

116 Actin Binding Rho Activating Protein *abra* **12.611** 5.415

117 CMRF35-like molecule 3 *cd300ld3* 1.251 **3.896**

118 Fibrinogen Beta Chain (Variant 2) *fgb* **3.402** 1.235

119 Solute Carrier Family 3 Member 2 (Variant 3) *slc3a2* **58.096** 17.731

120 Progestin And AdipoQ Receptor Family Member 6 *paqr6* **3.574** 1.957

121 Growth Arrest And DNA Damage Inducible Gamma (Variant 4) *gadd45g*  **81.323** 33.078

122 Complement C7 (Variant 1) *c7* **2.348** 0.575

123 Ras Homolog Family Member U (Variant 1) *rhou* **2.949** 1.104

124 Ras Homolog Family Member U (Variant 2) *rhou* **2.949** 1.104

125 Solute Carrier Family 3 Member 2 (Variant 4) *slc3a2* **59.921** 18.510

126 Solute Carrier Family 3 Member 2 (Variant 5) *slc3a2* **51.338** 15.973

127 Diablo IAP-Binding Mitochondrial Protein (Variant 2) *diablo* **3.344** 0.408

128 Complement C7 (Variant 2) *c7* **4.633** 1.125

129 RAN Binding Protein 2 *ranbp2*  0.685 **1.603**

130 Suppressor Of Cytokine Signaling 3 (Variant 2) *socs3* **2.238** 0.795

131 Suppressor Of Cytokine Signaling 3 (Variant 3) *socs3* **2.206** 0.784

132 Solute Carrier Family 3 Member 2 (Variant 6) *slc3a2* **60.648** 18.595

133 Suppressor Of Cytokine Signaling 3 (Variant 4) *socs3* **1.438** 0.605

134 Suppressor Of Cytokine Signaling 3 (Variant 5) *socs3* **1.438** 0.605

135 Solute Carrier Family 3 Member 2 (Variant 7) *slc3a2*  **62.066** 18.524

136 Myosin Regulatory Light Chain Interacting Protein *mylip* **1.148** 0.567

137 Cysteine And Serine Rich Nuclear Protein 1 *csrnp1* **2.323** 1.305

138 BICD Cargo Adaptor 2 *bicd2* 1.428 **2.568**

139 Insulin Like Growth Factor 1 *igf1* **35.674** 10.798

140 Myelin Associated Glycoprotein *mag* **0.865** 0.470

141 Growth Arrest And DNA Damage Inducible Gamma (Variant 5) *gadd45g* **90.043** 37.788

142 Low Density Lipoprotein Receptor (Variant 2) *ldlr* **0.358** 0.107

143 Fibrinogen Alpha Chain *fga* **3.035** 1.167
