# Supplementary material for: Combinatorial metabolomic and transcriptomic analysis of muscle growth in hybrid striped bass (female white bass Morone chrysops x male striped bass M. saxatilis)
Source: BMC Genomics. 2024 Jun 10;25:580. doi: 10.1186/s12864-024-10325-y (PMC11165755; doi:10.1186/s12864-024-10325-y)
Supplement: Supplementary file 11 — Supplementary Material 11. [file 12864_2024_10325_MOESM11_ESM.docx]

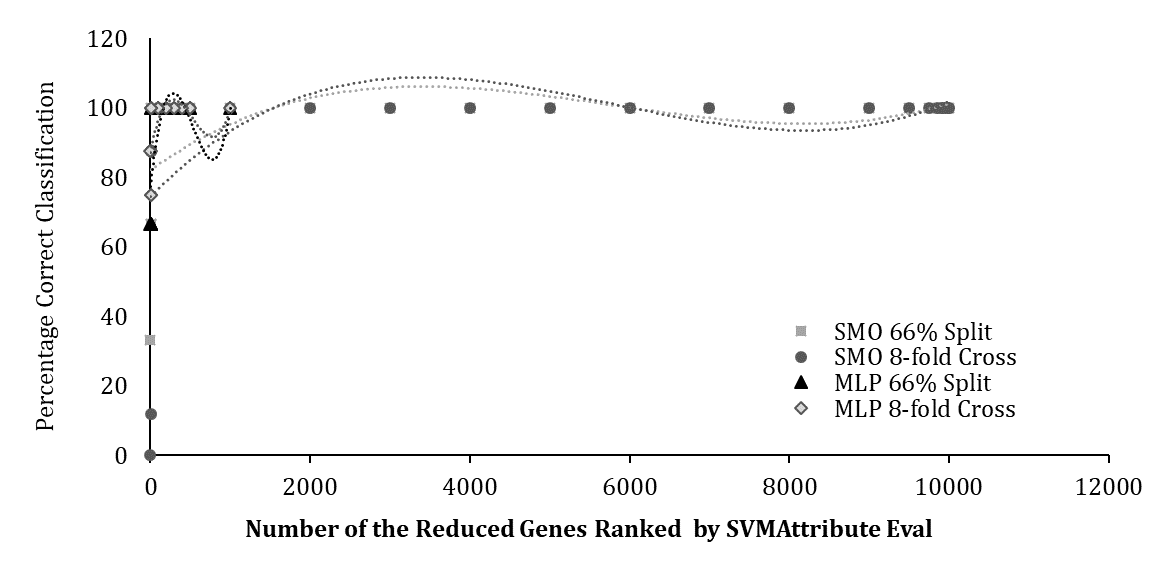


**Additional File 11 (Supplemental Figure 7).** Performance of Sequential Minimal Optimization Support Vector Machines (SMO) underfitting models used to evaluate expression of informative gene transcripts in hybrid striped bass white muscle. The SMO models were used to classify fish into good- and poor-growth groups and Fragments per Kilobase of Transcript per Million Mapped Reads (FPKM) expression values were used as input for each of the genes. Genes were ranked by importance during classification using SVMAttributeEval. The graph shows cross-validation performance of different models as percentage of correctly classified instances on the y-axis when the top 10000 ranked genes were used as baseline for SMO model performance and then the top highly ranked genes by SVMAttributeEval (10, 25, 50, 75, 100, 150, 250, 500, 1000, 2000, 3000, 4000, 5000, 6000, 7000, 8000, 9500, 9600, 9700, 9800, 9900, 9990, 9995, 9996, 9997, 9998, and 9999 genes) were subsequently eliminated from the input list on the x-axis. Two cross-validation strategies were used to evaluate the model learning: (1) a percentage split whereby 66 % of the data were randomly selected and used to train the models and the remaining 34 % of the data were input as a cross-validation and (2) a 8-fold stratified hold out with n = 8 folds where one fold was used for cross-validation and n – 1 folds of the randomly reordered data set were used for training. Both classes (good- and poor-growth groups) were properly represented in the model training and cross-validation data sets. Data points were fit with a polynomial line of the third order (dashed lines) and show optimal model performance when the top 1000 to 10000 genes were included in the models; model performance declined when 150 or more gene input values were omitted, indicating underfitting of the classification.
