# Supplementary material for: Combinatorial metabolomic and transcriptomic analysis of muscle growth in hybrid striped bass (female white bass Morone chrysops x male striped bass M. saxatilis)
Source: BMC Genomics. 2024 Jun 10;25:580. doi: 10.1186/s12864-024-10325-y (PMC11165755; doi:10.1186/s12864-024-10325-y)
Supplement: Supplementary file 12 — Supplementary Material 12. [file 12864_2024_10325_MOESM12_ESM.docx]

**Additional File 12 (Supplemental Table 5).** List of the top 150 most important genes in white skeletal muscle of hybrid striped bass that were chosen for the pathway analysis (genes are ranked based on machine learning SVMAttributeEval). Differences in elevated expression level between the two growth groups (i.e., poor- and good-growth) is expressed as the average FPKM value (Fragments per Kilobase of Transcript per Million Mapped Reads). Up-regulated genes between the two growth groups are indicated in bold-faced text. The variant designation indicates that several genes were identified more than once, such that different *de novo* transcript assembles corresponding to the same gene were identified (e.g., spliced variants or allelic variants originating from the striped bass and white bass genomes as expressed in the hybrid striped bass).

Average FPKM

Poor- Good-

Rank and Gene Name Gene Symbol Growth Growth

1 RUN Domain Containing 3A *rundc3a* **5.779** 3.751

2 Cyclic AMP-responsive element-binding protein 3-like protein 4 (Variant 1) *creb3l4*  0.494 **1.221**

3 Anti-Silencing Function 1B Histone Chaperone (Variant 1) *asf1b* 0.320 **0.773**

4 Matrix Metallopeptidase 17 (Variant 1) *mmp17* **2.056** 0.885

5 Fragile X Mental Retardation 1 (Variant 1) *fmr1* 1.936 **2.565**

6 Anti-Silencing Function 1B Histone Chaperone (Variant 2) *asf1b* 0.147 **0.485**

7 Proliferation-Associated 2G4 (Variant 1) *pa2g4* 11.019 **16.897**

8 TRNA-YW Synthesizing Protein 3 Homolog (Variant 1) *tyw3* 1.599 **2.439**

9 Cyclic AMP-responsive element-binding protein 3-like protein 4 (Variant 2) *creb3l4* 0.322 **0.807**

10 Fragile X Mental Retardation 1 (Variant 2) *fmr1* 1.067 **1.420**

11 ADAM Metallopeptidase Domain 10 *adam10* 0.697 **0.790**

12 PWWP Domain Containing 2B *pwwp2b* 1.878 **2.450**

13 Coiled-Coil Domain Containing 88A *ccdc88a* **0.111** 0.073

14 Slit Guidance Ligand 2 *slit2*  0.209 **0.402**

15 Clustered Mitochondria Homolog (Variant 1) *cluh* 3.435 **5.123**

16 STE20-Related Kinase Adaptor Alpha (Variant 1) *strada* 0.776 **1.108**

17 Anti-Silencing Function 1B Histone Chaperone (Variant 3) *asf1b* 0.147 **0.485**

18 TRNA-YW Synthesizing Protein 3 Homolog (Variant 2) *tyw3* 0.513 **0.783**

19 Zinc Finger Protein 675 *znf675* 0.495 **0.783**

20 STE20-Related Kinase Adaptor Alpha (Variant 2) *strada* 0.776 **1.108**

21 Angiotensin II Receptor Type 2 (Variant 1) *agtr2* **1.260** 0.614

22 Clustered Mitochondria Homolog (Variant 2) *cluh* 3.435 **5.123**

23 SNF-related serine/threonine-protein kinase-like (Variant 1) *snrk* **0.853** 0.455

24 NudE Neurodevelopment Protein 1 (Variant 1) *nde1* 3.397 **4.348**

25 Regulator Of G Protein Signaling 13 *rgs13* **3.866** 0.579

26 U2 Small Nuclear RNA Auxiliary Factor 2 *u2af2* 2.433 **3.890**

27 REM2 And RAB Like Small GTPase 1 *rsg1* 0.401 **0.562**

28 BCL2 Like 1 *bcl2l1* 0.610 **0.929**

29 Splicing Factor 1 (Variant 1) *sf1* 2.907 **4.681**

30 Cysteine Rich Transmembrane BMP Regulator 1 *crim1* **2.284** 1.566

31 Proliferation-Associated 2G4 (Variant 2) *pa2g4* 9.802 **15.013**

32 NudE Neurodevelopment Protein 1 (Variant 2) *nde1* 1.411 **1.736**

33 Dual Specificity Tyrosine Phosphorylation Regulated Kinase 2 (Variant 1) *dyrk2* 7.261 **11.763**

34 Solute Carrier Family 25 Member 32 *slc25a32* 1.236 **1.655**

35 Dual Specificity Tyrosine Phosphorylation Regulated Kinase 2 (Variant 2) *dyrk2* 7.261 **11.763**

36 Hepatitis A Virus Cellular Receptor 2 *havcr2* 0.211 **1.404**

37 Splicing Factor 1 (Variant 2) *sf1* 2.936 **4.728**

38 Kelch Like Family Member 20 *klhl20* 0.219 **0.362**

39 Conserved Helix-Loop-Helix Ubiquitous Kinase (Variant 1) *chuk* 0.388 **0.693**

40 Inter-Alpha-Trypsin Inhibitor Heavy Chain Family Member 6 *itih6* **42.895** 28.646

41 PDS5 Cohesin Associated Factor A (Variant 1) *pda5a* 1.547 **2.540**

42 Metastasis Associated 1 Family Member 2 (Variant 1) *mta2* 1.115 **1.737**

43 Clathrin Interactor 1 *clint1* 1.754 **2.354**

44 SEC23 Interacting Protein *sec23ip* 0.337 **0.541**

45 C-X-C Motif Chemokine Receptor 4 (Variant 1) *cxcr4* **4.881** 1.609

46 Metastasis Associated 1 Family Member 2 (Variant 2) *mta2* 1.082 **1.679**

47 Janus Kinase 2 *jak2* **0.609** 0.497

48 Carbamoyl-Phosphate Synthetase 2 (Variant 1) *cad* 0.164 **0.369**

49 Solute Carrier Family 37 Member 2 *slc37a2* **0.332** 0.218

50 PDS5 Cohesin Associated Factor A (Variant 2) *pda5a* 0.919 **1.508**

51 Metastasis Associated 1 Family Member 2 (Variant 3) *mta2* 1.222 **1.941**

52 Conserved Helix-Loop-Helix Ubiquitous Kinase (Variant 2) *chuk* 0.207 **0.356**

53 Anti-Silencing Function 1B Histone Chaperone (Variant 4) *asf1b* 0.526 **1.191**

54 Metastasis Associated 1 Family Member 2 (Variant 4) *mta2* 1.069 **1.697**

55 WD Repeat Domain 46 (Variant 1) *wdr46* 0.862 **1.252**

56 Microtubule Affinity Regulating Kinase 3 *mark3* 1.920 **2.645**

57 Collagen Type XX Alpha 1 Chain *col20a1* **0.465** 0.258

58 Myocyte Enhancer Factor 2D *mef2d* 0.744 **1.048**

59 Serine/Arginine Repetitive Matrix 2 *srrm2* 1.681 **2.498**

60 CD22 Molecule *cd22* **1.138** 0.511

61 RAN Binding Protein 10 *ranbp10* 1.715 **2.459**

62 F-Box And Leucine Rich Repeat Protein 7 *fbxl7* **0.261** 0.181

63 U-Box Domain Containing 5 (Variant 1) *ubox5* 0.442 **0.807**

64 C-X-C Motif Chemokine Receptor 4 (Variant 2) *cxcr4* **2.834** 0.949

65 GDP Dissociation Inhibitor 1 *gdi1* **3.495** 2.451

66 Carbamoyl-Phosphate Synthetase 2 (Variant 2) *cad* 0.099 **0.201**

67 Nuclear Apoptosis Inducing Factor 1 *naif1* 0.231 **0.408**

68 WD Repeat Domain 46 (Variant 2) *wdr46* 0.876 **1.272**

69 CRK Like Proto-Oncogene, Adaptor Protein crkl 0.251 **0.399**

70 Growth Arrest And DNA Damage Inducible Beta (Variant 1) *gadd45b* **2.413** 0.975

71 DEAH-Box Helicase 8 *dhx8* 1.327 **3.003**

72 G Protein-Coupled Receptor 68 (Variant 1) *gpr68* 1.476 **2.092**

73 ATPase Family, AAA Domain Containing 2B (Variant 1) *atad2b* 0.245 **0.362**

74 Vacuolar Protein Sorting 13 Homolog B *vps13b* 0.168 **0.290**

75 Methylenetetrahydrofolate Reductase (Variant 1) *mthfr* 9.215 **11.999**

76 Carbamoyl-Phosphate Synthetase 2 (Variant 3) *cad* 0.109 **0.226**

77 Ras Homolog Family Member U *rhou* **2.914** 1.222

78 Cell Division Cycle 73 (Variant 1) *cdc73* 1.690 **2.454**

79 U-Box Domain Containing 5 (Variant 2) *ubox5* 0.447 **0.816**

80 Ral Guanine Nucleotide Dissociation Stimulator Like 1 (Variant 1) *rgl1* **3.043** 1.313

81 SMG1, Nonsense Mediated MRNA Decay Associated (Variant 1) *smg1* 0.375 **0.568**

82 Zinc Finger Protein 644 *znf644* 0.248 **0.409**

83 U-Box Domain Containing 5 (Variant 3) *ubox5* 0.393 **0.701**

84 Methylenetetrahydrofolate Reductase (Variant 2) *mthfr* 5.192 **6.804**

85 Mitochondrial Ribosomal Protein L42 *mrpl42*  2.354 **3.147**

86 Musashi RNA Binding Protein 1 (Variant 1) *msi1* **21.721** 12.383

87 Zinc Finger Protein 318 *znf318* 0.564 **0.721**

88 Ral Guanine Nucleotide Dissociation Stimulator Like 1 (Variant 2) *rgl1* **1.674** 0.723

89 U-Box Domain Containing 5 (Variant 4) *ubox5* 0.396 **0.706**

90 Cysteine Rich Angiogenic Inducer 61 *cyr61* 2.262 **2.856**

91 ECSIT Signalling Integrator (Variant 1) *ecsit*  1.575 **2.150**

92 G Protein-Coupled Receptor 68 (Variant 2) *gpr68* 1.040 **1.474**

93 Clustered Mitochondria Homolog (Variant 3) *cluh* 2.591 **3.090**

94 H2.0 Like Homeobox *hlx* 0.363 **0.931**

95 Enhancer Of mRNA Decapping 3 (Variant 1) *edc3* 0.149 **0.334**

96 Deoxyhypusine Hydroxylase (Variant 1) *dohh* 2.891 **3.750**

97 Progestin And AdipoQ Receptor Family Member 7 *paqr7* **0.321** 0.163

98 MAP7 Domain Containing 1 (Variant 1) *map7d1* **4.683** 2.706

99 Deoxyhypusine Hydroxylase (Variant 2) *dohh* 2.891 **3.750**

100 Chromosome 16 Open Reading Frame 72 (Variant 1) *c16orf72* 4.749 **6.108**

101 MAP7 Domain Containing 1 (Variant 2) *map7d1* **4.683** 2.706

102 Musashi RNA Binding Protein 1 (Variant 2) *msi1* **4.717** 2.792

103 Transmembrane Protein 131 *tmem131* 4.146 **5.501**

104 Growth Arrest And DNA Damage Inducible Beta (Variant 2) *gadd45b* **2.302** 0.930

105 Hook Microtubule Tethering Protein 2 (Variant 1) *hook2* 2.727 **3.431**

106 SNF-related serine/threonine-protein kinase-like (Variant 2) *snrk* **0.477** 0.268

107 Enhancer Of MRNA Decapping 3 (Variant 2) *edc3* 0.149 **0.334**

108 Transmembrane 9 Superfamily Member 1 *tm9sf1* 0.287 **0.497**

109 CREB Regulated Transcription Coactivator 1 *crtc1* 0.947 **1.626**

110 ARP8 Actin Related Protein 8 Homolog *actr8* 0.243 **0.445**

111 Serine/Threonine Kinase 17a *stk17a* **0.568** 0.415

112 Chromosome 12 Open Reading Frame 49 *cunh12orf49* 0.203 **0.296**

113 TRNA-YW Synthesizing Protein 3 Homolog (Variant 3) *tyw3* 0.449 **0.630**

114 Metastasis Associated 1 Family Member 2 (Variant 5) *mta2* 1.357 **2.442**

115 Serine And Arginine Repetitive Matrix 1 *srrm1* 2.998 **4.651**

116 Cell Division Cycle 73 (Variant 2) *cdc73* 1.690 **2.454**

117 CCAAT/Enhancer Binding Protein Beta (Variant 1) *cebpb* **3.408** 0.884

118 Metastasis Associated 1 Family Member 2 (Variant 6) *mta2* 1.387 **2.496**

119 SDS3 Homolog, SIN3A Corepressor Complex Component *suds3* 1.831 **2.946**

120 CCAAT/Enhancer Binding Protein Beta (Variant 2) *cebpb* **3.408** 0.884

121 ATPase Family, AAA Domain Containing 2B (Variant 2) *atad2b* 0.170 **0.237**

122 ECSIT Signalling Integrator (Variant 2) *ecsit* 2.321 **3.148**

123 WD Repeat Domain 46 (Variant 3) *wdr46*  0.823 **1.223**

124 Chromosome 16 Open Reading Frame 72 (Variant 2) *c16orf72* 4.749 **6.108**

125 WD Repeat Domain 46 (Variant 4) *wdr46* 0.778 **1.155**

126 Phosphoenolpyruvate Carboxykinase 1 *pck1* **0.470** 0.094

127 Spindlin 1 (Variant 1) *spin1* 4.926 **8.031**

128 SMG1, Nonsense Mediated MRNA Decay Associated (Variant 2) *smg1* 0.643 **0.962**

129 Ankyrin Repeat And Zinc Finger Domain Containing 1 *ankzf1* 0.964 **1.447**

130 Zinc Finger And BTB Domain Containing 33 *zbtb33* 0.234 **0.340**

131 Metastasis Associated 1 Family Member 2 (Variant 7) *mta2* 0.818 **1.472**

132 Angiotensin II Receptor Type 2 (Variant 2) *agtr2* **1.294** 0.690

133 Hook Microtubule Tethering Protein 2 (Variant 2) *hook2* 2.300 **3.143**

134 CDK5 Regulatory Subunit Associated Protein 2 *cdk5rap2* **4.763** 2.954

135 Snf2 Related CREBBP Activator Protein *srcap*  1.974 **3.071**

136 Matrix Metallopeptidase 17 (Variant 2) *mmp17*  **0.298** 0.144

137 Catenin Delta 1 *ctnnd1* 0.774 **0.871**

138 Phosphatidylglycerophosphate Synthase 1 *pgs1* 2.144 **3.108**

139 Transmembrane Protein 183A *tmem183a* 1.164 **1.490**

140 Glucosidase Alpha, Acid *gaa* **7.034** 5.655

141 Aggrecan *can* **0.565** 0.320

142 Spindlin 1 (Variant 2) *spin1* 4.926 **8.031**

143 NLR Family CARD Domain Containing 3 *nlrc3* 0.546 **0.700**

144 Exocyst Complex Component 2 *exoc2* 0.362 **0.587**

145 Kelch Like Family Member 40 *klhl40* 6.481 **8.428**

146 Metastasis Associated 1 Family Member 2 (Variant 8) *mta2* 1.631 **2.562**

147 Leucine Rich Repeats And Calponin Homology Domain Containing 1 *lrch1* 14.501 **16.012**

148 Beta-1,3-Glucuronyltransferase 1 *b3gat1* 0.522 **0.743**

149 Serine And Arginine Rich Splicing Factor 10 *srsf10* 3.309 **5.113**

150 Thyroid Hormone Receptor Interactor 11 *trip11* 1.164 **2.162**
