# Supplementary material for: Combinatorial metabolomic and transcriptomic analysis of muscle growth in hybrid striped bass (female white bass Morone chrysops x male striped bass M. saxatilis)
Source: BMC Genomics. 2024 Jun 10;25:580. doi: 10.1186/s12864-024-10325-y (PMC11165755; doi:10.1186/s12864-024-10325-y)
Supplement: Supplementary file 14 — Supplementary Material 14. [file 12864_2024_10325_MOESM14_ESM.docx]

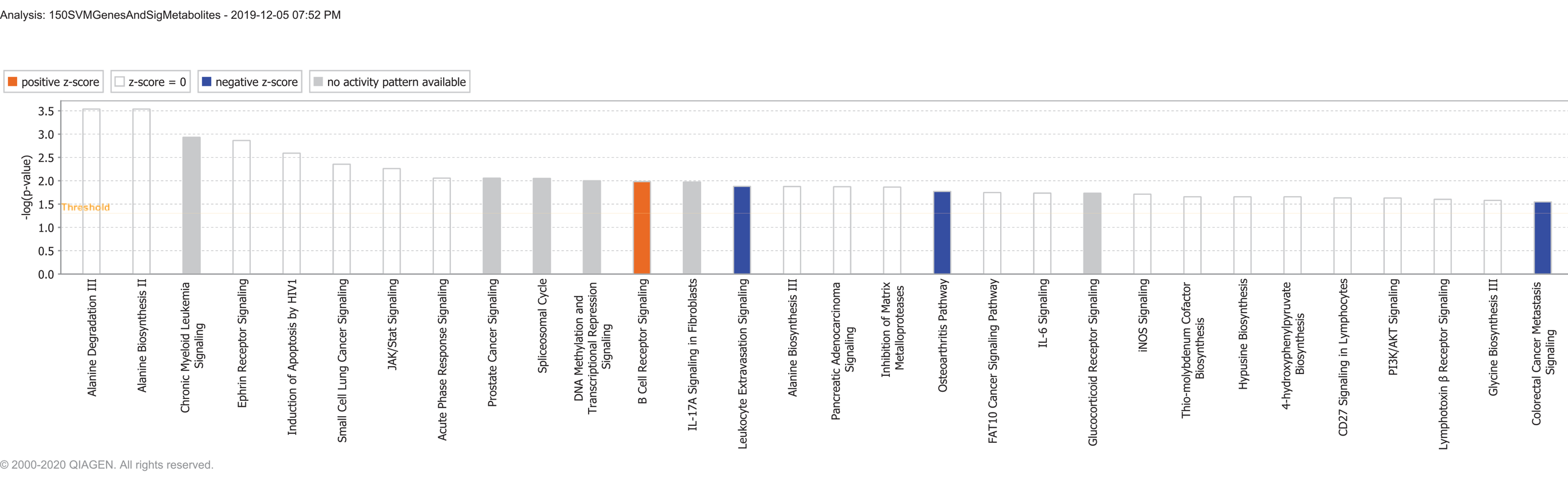


**Additional File 14 (Supplemental Figure 9).** Bar chart of significantly-enriched Qiagen Ingenuity Pathway Analysis (IPA) Canonical Pathways (The Fisher's Exact Test *p* ≤ 0.05) from the core analysis of 150 expressed genes ranked by SVMAttributeEval (WEKA machine learning) and 469 metabolites for hybrid striped bass muscle. Each bar represents a canonical pathway *p*-value on a negative logarithmic scale, such that taller bars are more significant than shorter bars. Bars are shaded according to their z-score activity predictions: (*orange*) pathways with positive z-scores (up-regulated in fish from the good-growth group); (*blue*) pathways with negative z-scores (down-regulated in fish from the good-growth group); (*white*) pathways with a z-score of 0, indicating that differential gene or metabolite expression data did not allow for a clear determination of directional activity prediction (i.e., the weight of evidence for predicted activation is equivalent to that of inhibition); and (*grey*) pathways for which no activity predictions could be accurately concluded due to lack of current information in the Qiagen Ingenuity Knowledge Base. Image was created using IPA (Qiagen, Germantown, MD, USA).
