# Supplementary material for: Combinatorial metabolomic and transcriptomic analysis of muscle growth in hybrid striped bass (female white bass Morone chrysops x male striped bass M. saxatilis)
Source: BMC Genomics. 2024 Jun 10;25:580. doi: 10.1186/s12864-024-10325-y (PMC11165755; doi:10.1186/s12864-024-10325-y)
Supplement: Supplementary file 16 — Supplementary Material 16. [file 12864_2024_10325_MOESM16_ESM.docx]

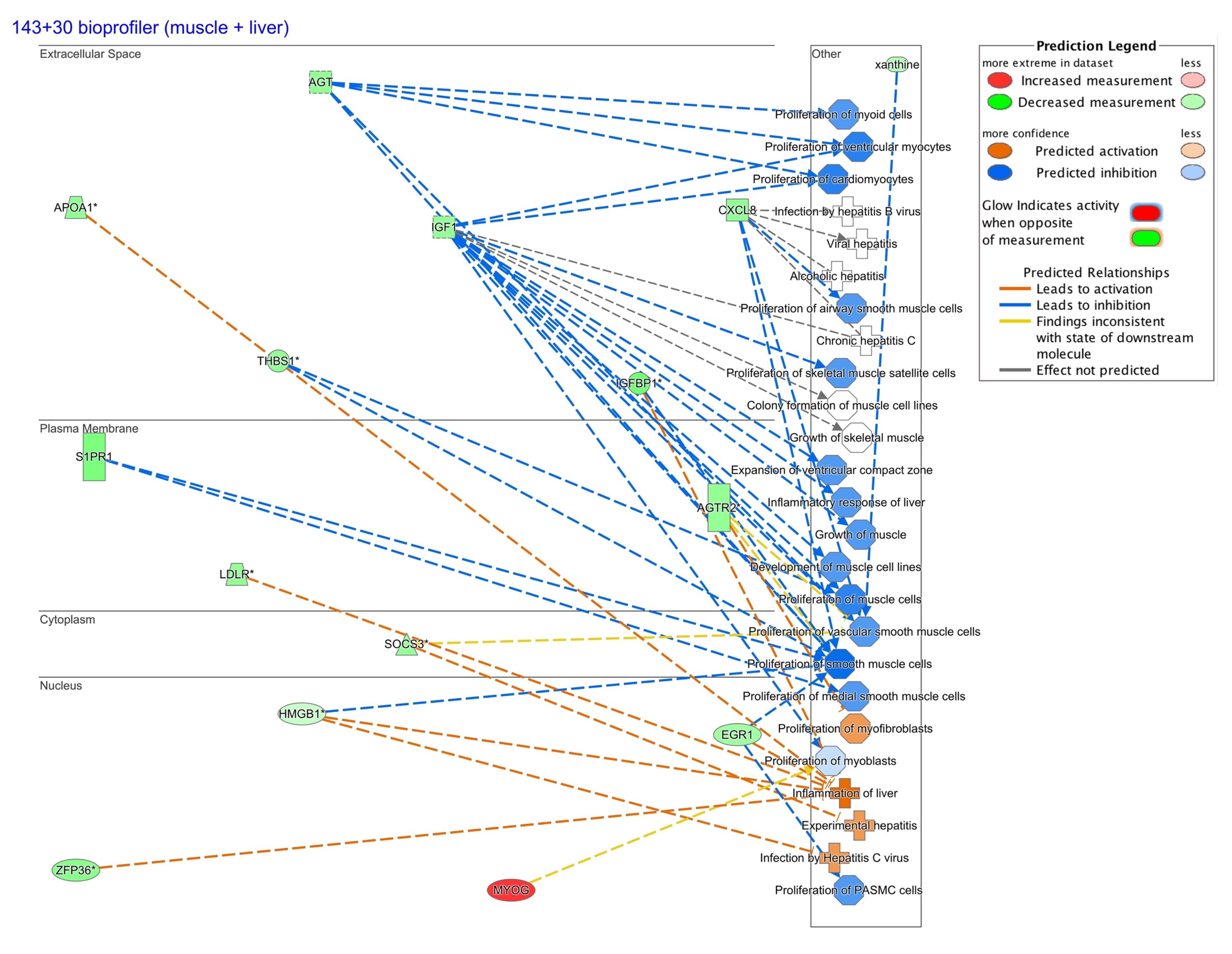


**Additional File 16 (Supplemental Figure 11).** BioProfiler pathway functional analysis in muscle of hybrid striped bass based on metabolites and genes identified by inferential statistics (FDR, q < 0.05). The network was generated based on measured gene expression and metabolite values that were predicted to inhibit (*blue*) or activate cell pathways (*orange*) in muscle of fish from the good-growth group relative to the poor-growth group. Down-regulation (*green*) refers to lower gene expression or metabolite levels measured in muscle of fish from the good-growth group relative to the poor-growth group, while up-regulation (*red*) refers to increased expression levels measured in muscle of fish from the good-growth group relative to the poor-growth group. Connections between molecules and pathway functions have previously published relationships in the literature. Arrows indicate activation and perpendicular lines indicate inhibition of effect; (*orange*) and (*blue*) lines indicate agreement and (*yellow*) lines indicate disagreement with previously published literature; (*grey*) lines indicate relationships that could not be accurately concluded due to lack of current information. Image was created using Ingenuity Pathway Analysis BioProfiler Analysis (Qiagen IPA, Germantown, MD, USA).
