# Supplementary material for: Combinatorial metabolomic and transcriptomic analysis of muscle growth in hybrid striped bass (female white bass Morone chrysops x male striped bass M. saxatilis)
Source: BMC Genomics. 2024 Jun 10;25:580. doi: 10.1186/s12864-024-10325-y (PMC11165755; doi:10.1186/s12864-024-10325-y)
Supplement: Supplementary file 17 — Supplementary Material 17. [file 12864_2024_10325_MOESM17_ESM.docx]

**Additional File 17 (Supplemental Figure 12).** B cell receptor signaling pathway significantly enriched (Fisher's Exact Test, *p* < 0.05) in muscle of hybrid striped bass based on metabolites and genes ranked by machine learning (SVMAttributeEval). Measured gene expression and metabolite values were predicted to inhibit apoptosis (*blue*) and activate gene transcription and leading to cell proliferation (*orange*) in muscle of fish from the good-growth group. Down-regulation (*green*) refers to lower gene expression levels measured in muscle of fish from the good-growth group relative to the poor-growth group, while up-regulation (*red*) refers to increased expression levels measured in muscle of fish from the good-growth group relative to the poor-growth group. Image was created using Ingenuity Pathway Analysis Core Analysis Molecule Activity Predictor (MAP) (Qiagen IPA, Germantown, MD, USA).
