# Supplementary material for: Combinatorial metabolomic and transcriptomic analysis of muscle growth in hybrid striped bass (female white bass Morone chrysops x male striped bass M. saxatilis)
Source: BMC Genomics. 2024 Jun 10;25:580. doi: 10.1186/s12864-024-10325-y (PMC11165755; doi:10.1186/s12864-024-10325-y)
Supplement: Supplementary file 18 — Supplementary Material 18. [file 12864_2024_10325_MOESM18_ESM.docx]

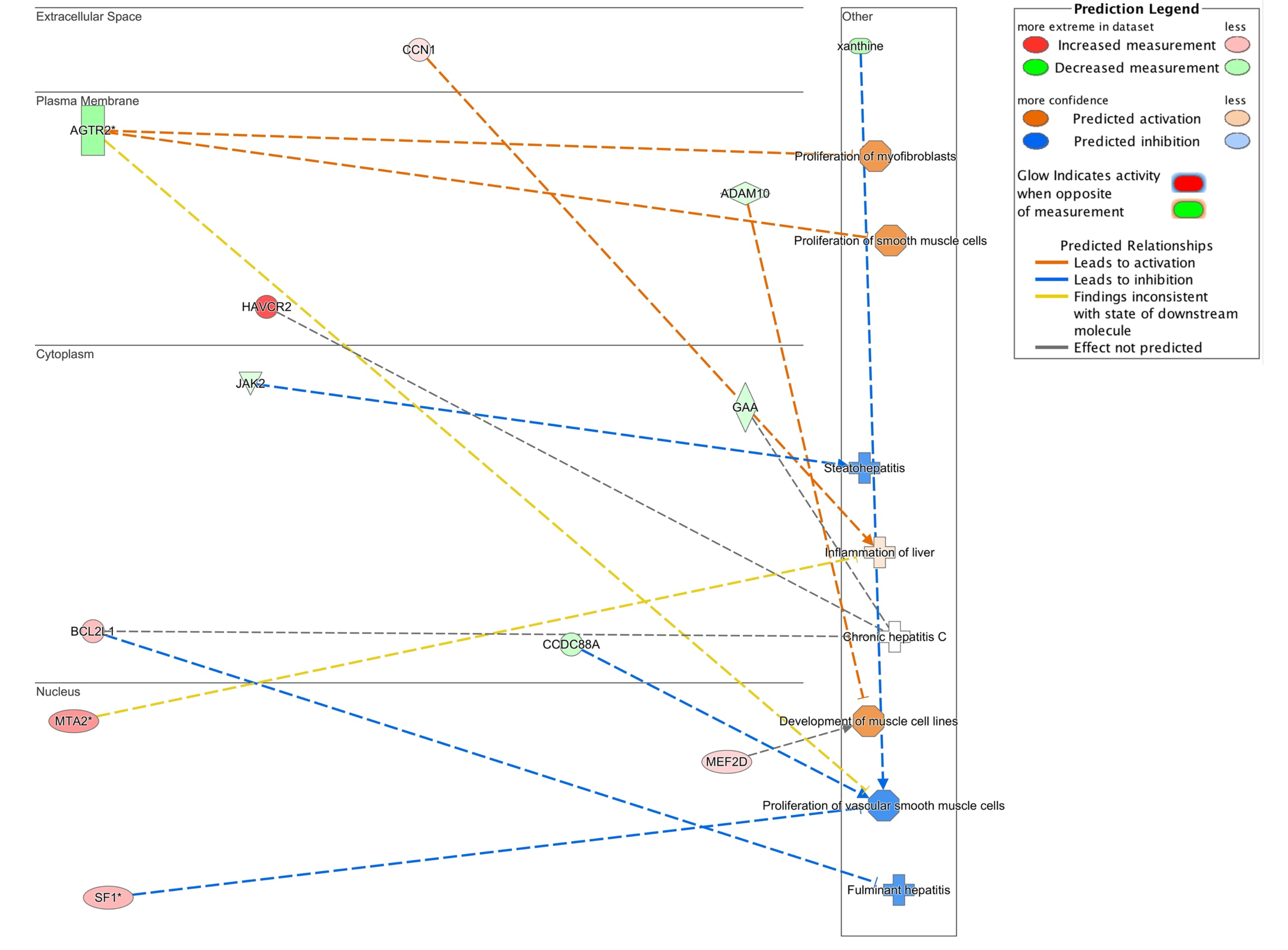


**Additional File 18 (Supplemental Figure 13).** BioProfiler pathway functional analysis in muscle of hybrid striped bass based on metabolites and genes ranked by machine learning (SVMAttributeEval). The network was generated based on measured gene expression and metabolite values that were predicted to inhibit (*blue*) or activate cell pathways (*orange*) in muscle of fish from the good-growth group relative to the poor-growth group. Down-regulation (*green*) refers to lower gene expression or metabolite levels measured in muscle of fish from the good-growth group relative to the poor-growth group, while up-regulation (*red*) refers to increased expression levels measured in muscle of fish from the good-growth group relative to the poor-growth group. Connections between molecules and pathway functions have previously published relationships in the literature. Arrows indicate activation and perpendicular lines indicate inhibition of effect; (*orange*) and (*blue*) lines indicate agreement and (*yellow*) lines indicate disagreement with previously published literature; (*grey*) lines indicate relationships that could not be accurately concluded due to lack of current information. Image was created using Ingenuity Pathway Analysis BioProfiler Analysis (Qiagen IPA, Germantown, MD, USA).
