# Supplementary material for: Combinatorial metabolomic and transcriptomic analysis of muscle growth in hybrid striped bass (female white bass Morone chrysops x male striped bass M. saxatilis)
Source: BMC Genomics. 2024 Jun 10;25:580. doi: 10.1186/s12864-024-10325-y (PMC11165755; doi:10.1186/s12864-024-10325-y)
Supplement: Supplementary file 22 — Supplementary Material 22. [file 12864_2024_10325_MOESM22_ESM.docx]

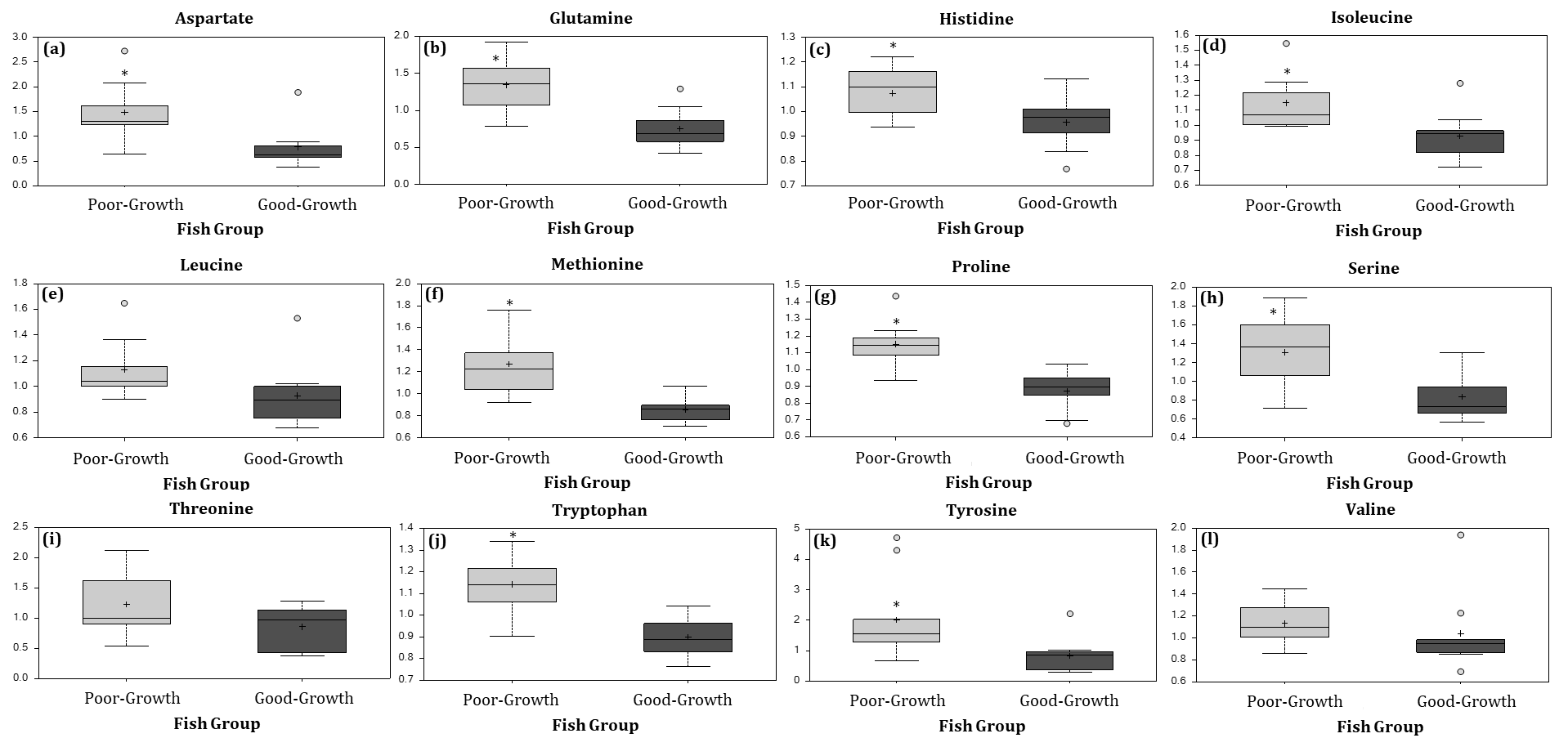


**Additional File 22 (Supplemental Figure 17).** Boxplots showing a number of amino acids (a) aspartate, (b) glutamine, (c) histidine, (d) isoleucine, (e) leucine, (f) methionine, (g) proline, (h) serine, (i) threonine, (j) tryptophan, (k) tyrosine, and (l) valine for scaled intensity ranges in liver of hybrid striped bass from the poor- and good- growth groups (* indicates *p* < 0.05; N=9/group). Median value is shown as the line through each box. Mean value is represented as the “+” symbol; potential outliers are indicated with a small circle. Upper and lower quartile ranges are the top and bottom of each box. Maximum and minimum distribution for each group is designated by the top and bottom whiskers.
