# Supplementary material for: Combinatorial metabolomic and transcriptomic analysis of muscle growth in hybrid striped bass (female white bass Morone chrysops x male striped bass M. saxatilis)
Source: BMC Genomics. 2024 Jun 10;25:580. doi: 10.1186/s12864-024-10325-y (PMC11165755; doi:10.1186/s12864-024-10325-y)
Supplement: Supplementary file 25 — Supplementary Material 25. [file 12864_2024_10325_MOESM25_ESM.docx]

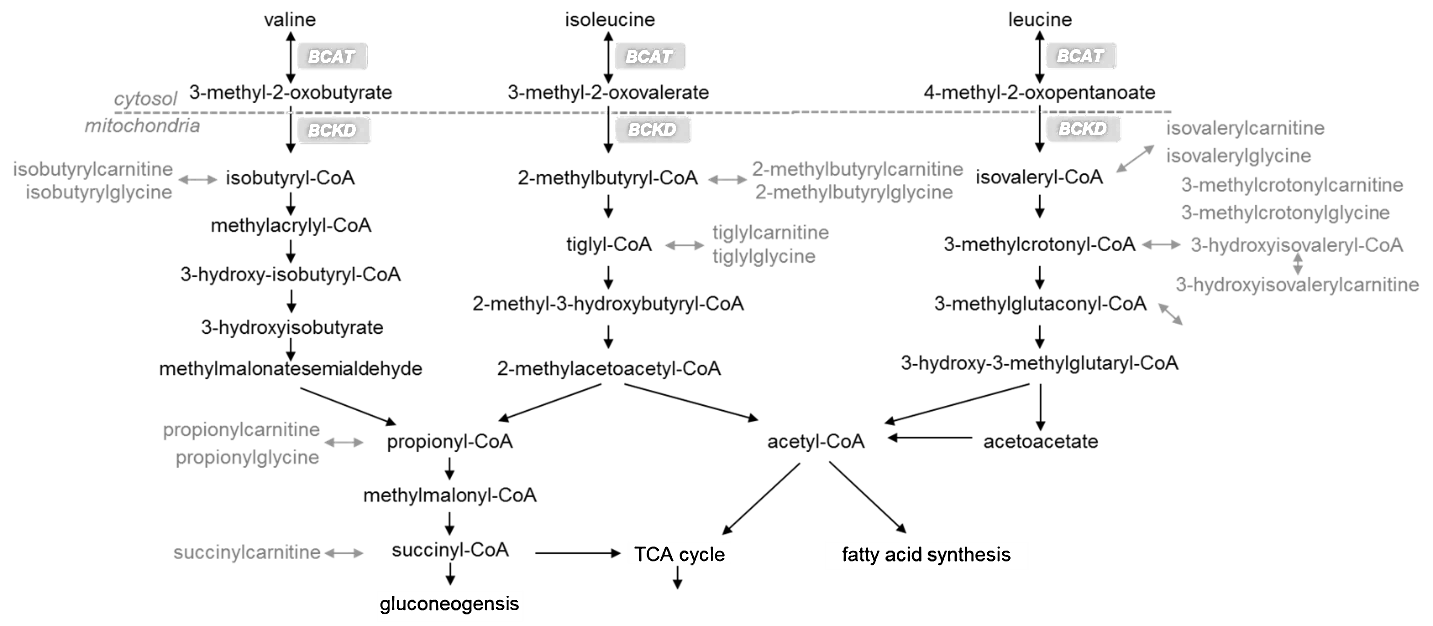


**Additional File 25 (Supplemental Figure 20).** Diagram of branched chain amino acid (BCAA; valine, isoleucine, leucine) catabolism pathways identified in hybrid striped bass liver. Catabolized BCAA may be assimilated for energy production through the citric acid cycle (TCA) in fish from the good-growth group.
