# Supplementary material for: Combinatorial metabolomic and transcriptomic analysis of muscle growth in hybrid striped bass (female white bass Morone chrysops x male striped bass M. saxatilis)
Source: BMC Genomics. 2024 Jun 10;25:580. doi: 10.1186/s12864-024-10325-y (PMC11165755; doi:10.1186/s12864-024-10325-y)
Supplement: Supplementary file 29 — Supplementary Material 29. [file 12864_2024_10325_MOESM29_ESM.docx]

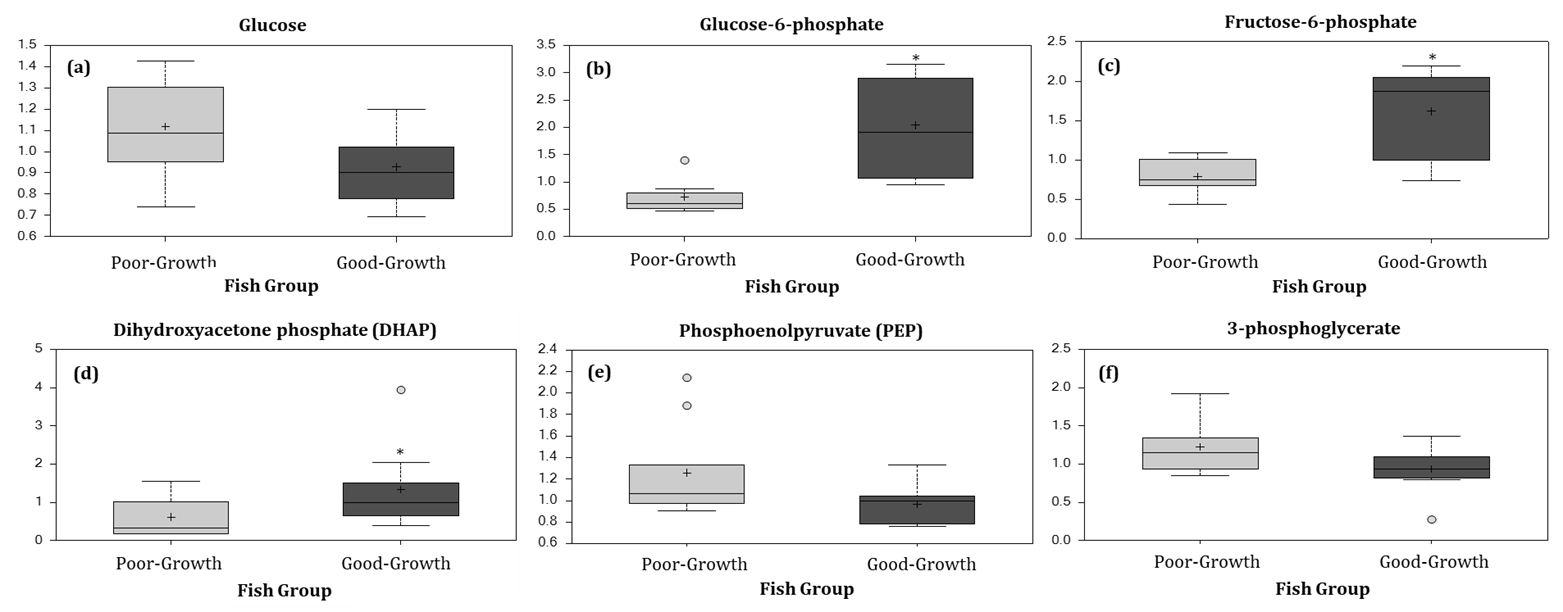


**Additional File 29 (Supplemental Figure 24).** Boxplots showing (a) glucose, (b) glucose-6-phosphate, (c) fructose-6-phosphate, (d) dihydroxyacetone phosphate (DHAP), (e) phosphoenolpyruvate (PEP), and (f) 3-phosphoglycerate for scaled intensity ranges in liver of hybrid striped bass from the poor- and good- growth groups (* indicates *p* < 0.05; N=9/group). Median value is shown as the line through each box. Mean value is represented as the “+” symbol; potential outliers are indicated with a small circle. Upper and lower quartile ranges are the top and bottom of each box. Maximum and minimum distribution for each group is designated by the top and bottom whiskers.
